# Optimization of crossing strategy based on the usefulness criterion in interpopulation crosses considering different marker effects among populations

**DOI:** 10.1007/s00122-025-04935-7

**Published:** 2025-06-20

**Authors:** Sei Kinoshita, Kengo Sakurai, Kosuke Hamazaki, Takahiro Tsusaka, Miki Sakurai, Kenta Shirasawa, Sachiko Isobe, Hiroyoshi Iwata

**Affiliations:** 1 Graduate School of Agricultural and Life Sciences, University of Tokyo, Tokyo, Japan; 2 RIKEN Center for Advanced Intelligence Project, Chiba, Japan; 3 TSUMURA & CO, Ibaraki, Japan; 4 Kazusa DNA Research Institute, Chiba, Japan

## Abstract

**Key message:**

Herein, a method has been proposed for selecting optimal cross pairs based on the genetic potential of progeny in interpopulation crosses, considering different genetic effects among populations.

**Abstract:**

In the breeding programs for self-pollinating plants, genetic improvements in multiple traits can be challenging when relying solely on a single biparental population, and interpopulation crosses are employed to integrate favorable alleles from multiple biparental populations to overcome this limitation. In this context, it is crucial to consider distinct genetic effects in different populations. In this study, we used a selection method based on the usefulness criterion (UC) to identify cross pairs suitable for interpopulation crosses. We expanded this approach to enhance breeding programs by accounting for varying genetic backgrounds within the genomic selection framework. Using the medicinal plant perilla as the study material, we performed simulations to compare the efficacy of selection based on the estimated genotypic values with that of selection based on UC. Our findings demonstrate that the proposed method is effective in facilitating the simultaneous improvement of multiple traits, particularly by considerably increasing the genetic gains among the top-performing individuals in the population. Furthermore, we provide guidelines for implementing interpopulation crosses, including recommendations for the optimal generation for crossing and an appropriate reference generation for calculating UC. The results obtained in this study offer valuable insights for small-scale breeding programs aimed at simultaneously enhancing multiple traits through interpopulation crosses and can be applied to a wide range of crops, including neglected and underutilized species.

**Supplementary Information:**

The online version contains supplementary material available at 10.1007/s00122-025-04935-7.

## Introduction

Genomic selection (GS) utilizes genome-wide marker information to predict the genetic potential of individuals and selects them based on predicted genotypic values (Meuwissen et al. 2001). Initially, GS was used in dairy cattle breeding (Hayes et al. 2009); however, more recently, it has been actively used in plant breeding (Bernardo and Yu 2007; Yamamoto et al. 2016; Mahadevaiah et al. 2021). GS has helped develop several breeding schemes tailored to the specific traits of different plant species. For wheat breeding, recurrent selection within a single biparental population is commonly employed (Bassi et al. 2016). The simulation studies of wheat breeding programs have typically incorporated strategies such as random mating, selfing, and the production of doubled haploids (DHs) (Daetwyler et al. 2015; Gaynor et al. 2017). However, these studies have generally focused on repeated mating or selfing within a single biparental population, with few considering using multiple parents as the initial crossing parents in the context of GS.

Many breeding programs have used multiparental populations throughout the history of plant breeding. The use of multiple parents is believed to increase genetic diversity and facilitate the development of varieties with superior traits (Arrones et al. 2020). Studies have focused on integrating multiple desirable genes into a single population by crossing different populations derived from various parental lines in a process known as gene pyramiding (Ramalingam et al. 2020). In genomic breeding using multiple parents or populations, it is crucial to consider the distinct marker effects of each parent or population. Here, ‘distinct marker effects’ refer to the genetic effects of differences in linkage patterns with quantitative trait loci (QTLs). Models for QTL analysis suitable for multiple populations with different genetic backgrounds have been developed, assuming that the effects and locations of QTLs may vary (Li et al. 2021). These models have been applied to various plants and are considered to enhance the detection power of QTLs (Mangino et al. 2022). To further explore the necessity of considering distinct marker effects (effects of QTLs) for each population, Bernardo (2008) suggested that QTL effects can be consistent across different populations if the populations share a common parent carrying a specific QTL allele. For example, when a specific resistance gene is introduced from a common parent, the effect of the corresponding QTL is likely to be consistent among the populations derived from that parent. However, in typical breeding populations, the parents often have different genetic backgrounds and have undergone different selection histories. Therefore, different QTLs are likely to segregate in different populations. This divergence in the segregation of QTLs among populations is more pronounced for complex traits controlled by many minor QTLs than for simple traits controlled by a few major QTLs. In marker-assisted recurrent selection, differences in QTL effects among populations are considered by repeatedly conducting genotyping, phenotyping, and constructing a selection index for each population (Koebner 2003).

Distinct marker effects within different populations are particularly advantageous for small-scale breeding programs. For example, the genetic traits of neglected underutilized species (NUS), which have recently gained attention because of their high nutritional value and adaptability to sustainable agriculture, can be improved using efficient breeding strategies, such as GS (Ye and Fan 2021). However, implementing large-scale breeding programs for NUS is challenging because of the limited genetic and economic resources available for such species. In such cases, a practical approach involves selecting a promising set of lines, creating multiple biparental populations, and subsequently combining these populations. It has been hypothesized that, in such small-scale breeding populations, allele effects vary based on the population (Maurer et al. 2017). In the present study, we focused on the medicinal plant red perilla, a type of NUS that has undergone small-scale breeding in multiple biparental populations. The parents of these multiple perilla populations included traditional varieties collected from different regions of Japan and existing red perilla medicinal cultivars. As the initial cross-parents had distinct genetic backgrounds, the genetic effects of markers within each biparental population were also expected to differ. These perilla populations exhibit superior characteristics in different traits, as they produce various bioactive compounds with medicinal value; however, there remains a pressing need to simultaneously improve the content of multiple bioactive compounds in these populations (Kinoshita et al. 2023). In the present study, we investigated multiple biparental populations with distinct marker effects in the context of GS to evaluate the effectiveness of interpopulation crosses in enhancing multiple traits.

In plant breeding programs, new varieties are developed through repeated crossings and selection cycles. Thus, it is essential not only to select superior individuals but also to identify optimal mating pairs that can produce high-performing progeny. Typically, mating individuals with high genotypic values ensures a high performance in the population mean of the progeny. However, in breeding, it is crucial to maintain not only a high progeny mean but also the ability to produce superior progeny over the long term (Sanchez et al. 2023). Selecting cross pairs capable of generating progeny with high genetic variance is necessary to sustain genetic gain over time.

In 1975, Schnell and Utz introduced the ‘usefulness criterion’ (UC) to measure the trait mean of the upper fraction of progeny from a given cross. UC is defined as $\mu =ih\sigma$, where $\mu$ is the mean breeding value of parents, $i$ is the selection intensity, $h$ is the square root of heritability, and $\sigma$ is the square root of genetic variance in progeny. The square root of heritability ($h$) can be assumed to be 1 when selection is based on genotypic values (Zhong and Jannink 2007). With the advent of high-density marker information, integrating genomic prediction (GP) with UC has become feasible, enabling the prediction of genetic variance in progeny using genotypic values. Initially, the genetic variance of the progeny was predicted by simulating their genomes (Iwata et al. 2013; Bernardo 2014; Mohammadi et al. 2015). However, as the number of potential crosses and progeny increase, the computational cost of the simulations becomes prohibitive. To address this issue, Lehermeier et al. (2017) proposed an analytical approach to calculate the genetic variance of the progeny using estimated marker effects and recombination rates. Further advancements have expanded this method beyond simple biparental crosses, improving the computational efficiency for more complex breeding designs, such as three- and four-way crosses (Allier et al. 2019; Danguy des Déserts et al. 2023). In these studies, breeding programs typically focused on developing recombinant inbred lines (RILs) or DHs through biparental crosses, with no examples of their application in interpopulation crosses.

In the present study, we integrated the methods developed by Lehermeier et al. (2017) and Danguy des Déserts et al. (2023) to compute the genetic variance of the progeny derived from crosses between multiple biparental populations with different marker effects. The computed genetic variance was then used as a criterion to select cross pairs. In this study, we aimed to extend the calculation of UC to cases where marker effects differ across multiple biparental populations under the assumption of a breeding scheme that seeks to improve multiple traits through crossing. In addition, we aimed to verify the effectiveness of the UC in such situations. Furthermore, we sought to propose guidelines for performing such interpopulation crosses. Therefore, we performed simulation-based analyses using data from two red perilla RIL populations, each exhibiting different traits. Specifically, we propose a method for selecting mating pairs using UC, which accounts for marker effects varying across populations. In addition, we identify the optimal generation at which these crosses must be performed to achieve the desired outcomes.

## Materials and methods

### Plant materials

The plant materials used in this study consisted of two breeding populations of the F_4_ generation from two-way crosses between red and green perilla (Kinoshita et al. 2023). These populations were generated by crossing ‘SekihoS8’ × st27 (S827) and ‘SekihoS8’ × st40 (S840). Hereafter, these populations will be referred to as S827 and S840, respectively. ‘SekihoS8’ is a representative variety of red perilla (*Perilla frutescens* var*. crispa* f*. purpurea*) and was provided by TSUMURA & CO. (Ibaraki, Japan). Moreover, st27 and st40 are green perilla (*P. frutescens Britton* var. *crispa* Decne.) lines obtained from germplasm collections managed by the Genebank of the National Agriculture and Food Research Organization, Japan. The S827 and S840 populations contained 298 and 297 individuals with different genotypes, respectively. Kinoshita et al. (2023) used three biparental populations, i.e., S827, S840, and S844. Owing to the low number of single nucleotide polymorphisms (SNPs) and low GP accuracy, S844 was excluded from further analyses in this study. Further details of these two populations have been described by Kinoshita et al. (2023).

### Reference genome construction

The DNA of ‘SekihoS8’ was extracted using the Genomic-tip kit (QIAGEN Technologies). Genome sequencing was performed using the Sequel II platform (Pacific Biosciences), and HiFi reads were generated with the CCS version 4.2.0 (Pacific Biosciences) software, requiring a minimum of three subreads per read. Genome assembly was performed using hifiasm v0.16.1 (Cheng et al. 2021), and candidate chloroplast and mitochondrial genome sequences were eliminated using the chloroplast genome sequence of *P. frutescens* (NC_030755.1) and mitochondrial genome sequences of *Scutellaria galericulata* and *Ballota nigra* (OX335799.1, OX344731.1, OX344732.1, and OX344733.1) as reference sequences. The resulting scaffolds were aligned to the previously reported genome sequence of the perilla cultivar ‘Hoko-3’ (Tamura et al. 2023) using the RaGOO tool (Alonge et al. 2019). Genome structure comparisons were performed using D-Genies (Cabanettes and Klopp 2018). Genome structure analyses using k-mers and telomere sequence detection were performed using Smudgeplot and tidk, respectively (Ranallo-Benavidez et al. 2020; De la Rosa and Mark 2023). Gene prediction was performed using the Helixer 0.3.2 (land_plant_v0.3_a_0080). h5) (Stiehler et al. 2021). The accuracy of the assembled genome and predicted gene sequences were evaluated using the benchmarking universal single-copy orthologs (BUSCO) v5.2.2 (obd10) tool (Simão et al. 2015).

### Genotype and phenotype data

Genotypic and phenotypic data were obtained using the methods described in Kinoshita et al. (2023), with a few modifications. In this section, we describe the differences from the published method and provide an overview of the data acquisition process. The F_4_ generations, S827 and S840, were phenotyped for two traits, perillaldehyde and rosmarinic acid. These are the main medicinal compounds in perilla and are widely used in traditional herbal medicine. A detailed description of the methods for measuring perillaldehyde and rosmarinic acid content has been provided previously (Kinoshita et al. 2023).

All 595 individuals from the F_4_ generations of S827 and S840 were genotyped by double-digest restriction site-associated DNA sequencing (dd-RAD-Seq) method (Shirasawa et al. 2016). The obtained reads were then mapped onto the assembled sequence of ‘SekihoS8’ using Bowtie2 (Langmead and Salzberg 2012), which differed from the reference sequence used in Kinoshita et al. (2023). The details of reference genome construction are described in the previous section. Following variant calling, quality control, and filtering based on minor allele frequency as described by Kinoshita et al. (2023), 1,951 SNPs were obtained across the entire population, with 692 SNPs with MAF > 0.05 in S827 and 1,439 SNPs with MAF > 0.05 in S840. There were 343 overlapping SNPs between S827 and S840, which were markers with MAF > 0.05 in both populations among a total of 1,951 SNPs. The genotype scores were coded as 0 for homozygous SNPs matching ‘SekihoS8,’ 1 for heterozygous SNPs, and 2 for homozygous SNPs matching the other parents.

We calculated the average heterozygosity and genomic heritability for each population using the aforementioned genotypic data. The heterozygosity was calculated using the following formula:

1 $$H=1-\frac{1}{L}{\sum }_{{l}=1}^{L}{\sum }_{i=1}^{2}{p}_{li}^{2}$$

where $H$ represents the average heterozygosity, $L$ is the total number of markers ($L=\text{1,951}$), and ${p}_{i}$ is the frequency of the $i$ ^th^ allele at the $l$ ^th^ locus.

Genomic heritability was calculated as ${h}^{2}=\frac{{\sigma }_{u}^{2}}{{\sigma }_{u}^{2}+{\sigma }_{e}^{2}}$, following the approach described in Kinoshita et al. (2023), where ${\sigma }_{u}^{2}$ is the estimated additive genetic variance, and ${\sigma }_{e}^{2}$ is the estimated error variance. ${\sigma }_{u}^{2}$ and ${\sigma }_{e}^{2}$ were estimated using the ‘EMM.cpp’ function in the ‘RAINBOWR’ package version 0.1.35 in R (Hamazaki and Iwata 2020). The calculated average heterozygosity and genomic heritability of each population are presented in Table 1.

**Table 1:** Average heterozygosity and genomic heritability of each population

	Average heterozygosity	Genomic heritability (perillaldehyde)	Genomic heritability (rosmarinic acid)
S827	0.18	0.76	0.40
S840	0.36	0.84	0.45

The population structures of S827 and S840 are shown in Fig. S1, wherein a Euclidean distance matrix was calculated using 1951 SNPs, followed by a principal coordinate analysis. All 595 individuals were plotted based on their first and second principal coordinates.

### GP model

**Fig. 1:**
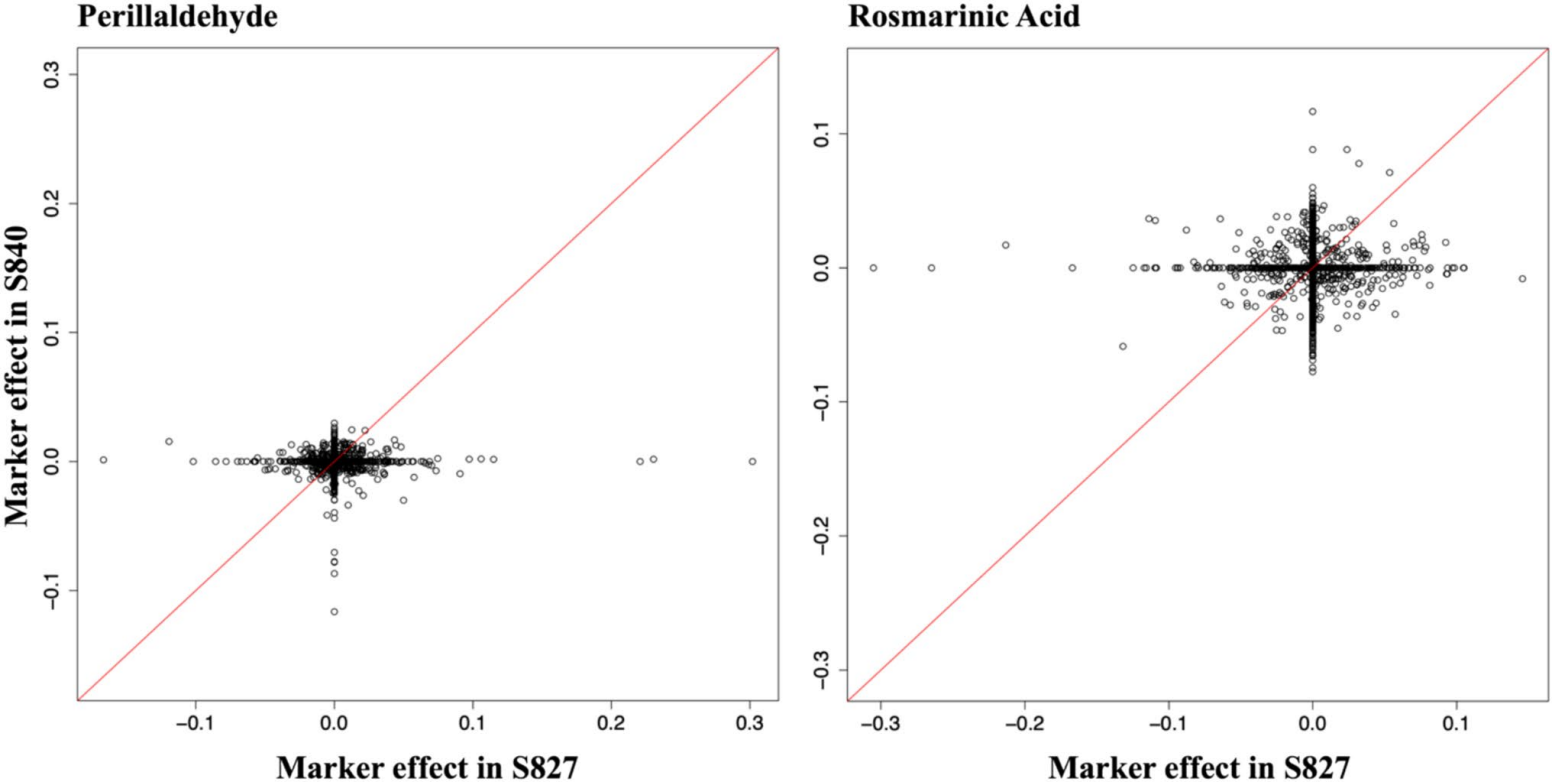
Estimated marker effects across 1,951 SNPs for each population and trait. The left panels displays estimated marker effects for perillaldehyde, while the right panel shows effects for rosmarinic acid. The x-axis represents marker effects estimated using S827, and the y-axis represents those estimated using S840 population

The BayesB model (Meuwissen et al. 2001) was used to estimate the marker effects for each trait and population. In this study, the GP model incorporates only additive effects. Although heterozygous markers were present in both the S827 and S840 populations (Table 1), dominant effects were excluded for two main reasons. First, our preliminary analyses demonstrated that models incorporating dominant effects failed to improve prediction accuracy and, in several instances, actually reduced it compared to additive-only models (Supplementary File 2). Second, our simulation assumes complete genetic fixation in the final generation, resulting in fully homozygous (inbred) individuals, rendering dominant effects irrelevant to the genetic gain. The Bayes B model is expressed as follows:

2 $${{\mathbf{y}}}_{pm}={\mu}_{pm}+{\mathbf{X}}_{p}{{\varvec{\beta}}}_{pm}+{{\varvec{\varepsilon}}}_{pm}$$

where $N$ is the number of individuals in one population, $L$ is the number of markers, ${{\mathbf{y}}}_{pm}$ is an $N\times 1$ vector representing phenotypic values for the $m$ ^th^ trait in the $p$ ^th^ population, ${\mu }_{pm}$ is the overall mean, ${\mathbf{X}}_{p}$ is an $N\times L$ matrix of marker genotypes for the $p$ ^th^ population, ${{\varvec{\beta}}}_{pm}$ is an $L\times 1$ vector corresponding to marker effects for the $m$ ^th^ trait and $p$ ^th^ population, and ${{\varvec{\varepsilon}}}_{pm}\sim N(0, \mathbf{I}{\sigma }_{e_{pm}}^{2})$ is an $N\times 1$ vector of errors, where ${\sigma }_{e}^{2}$ is the error variance. The Markov Chain Monte Carlo was run for 60,000 iterations, with the first 12,000 samples discarded as burn-in and a sampling interval (thinning) of 5. The BayesB model was implemented using the ‘BGLR’ function in the ‘BGLR’ package version 1.1.0 in R (Pérez and De Los Campos 2014). The estimated marker effects for all 1,951 SNPs are illustrated in Fig. 1 and S2. All individuals from both S827 and S840 populations were genotyped using 'SekihoS8' as the reference genome. Consequently, the nonzero marker effects estimated using S827 and S840 can be interpreted as effects contributed by the alternative parents, 'st27' and 'st40,’ respectively. Since both populations share the same reference sequence, marker effects estimated independently in each population are directly comparable on a common scale, with 'SekihoS8' effects serving as the baseline. This allows these marker effects to be considered absolute values rather than relative ones. The estimated marker effects were used in the subsequent simulations.

The rationale for considering interpopulation crosses based on marker effects estimated separately for each population is outlined below. S827 and S840 exhibited different patterns of marker effects for each trait. For perillaldehyde, many markers had favorable effects, whereas for rosmarinic acid, many markers showed negative effects in S827, however, demonstrated positive effects in S840. Table 2 presents the upper bounds of the attainable genotypic values, hereafter referred to as ‘ideal genotypic values,’ under both intra- and interpopulation selection scenarios. The ideal genotypic value for within-population selection represents the genotypic value achieved when all loci carry the favorable alleles from either 'SekihoS8' or 'st27' (or 'st40'). Conversely, the ideal genotypic value for selection across both populations represents the case where all loci carry the most favorable allele among 'SekihoS8', ‘st27,’ and 'st40.’ As demonstrated in Table 2, the ideal genotypic values for both traits were substantially higher when favorable alleles from both populations were combined. Specifically, interpopulation selection yielded a 1.43-fold increase in the ideal genotypic value for perillaldehyde compared to selection within S827 alone, and a 1.73-fold increase for rosmarinic acid compared to selection within S840 alone. These findings indicate that limiting selection to within each population could result in missing favorable alleles unique to other populations. Interpopulation crosses facilitate the combination of complementary favorable alleles from both populations, thereby maximizing potential genetic gain.

**Table 2:** Comparison of ideal genotypic values for each trait under two breeding scenarios: improvement within individual population versus integration of both populations

	Use only S827	Use only S840	Use both populations
Perillaldehyde	12.94	6.62	18.52
Rosmarinic acid	18.86	23.17	40.11

## Simulations

### Breeding program

**Fig. 2:**
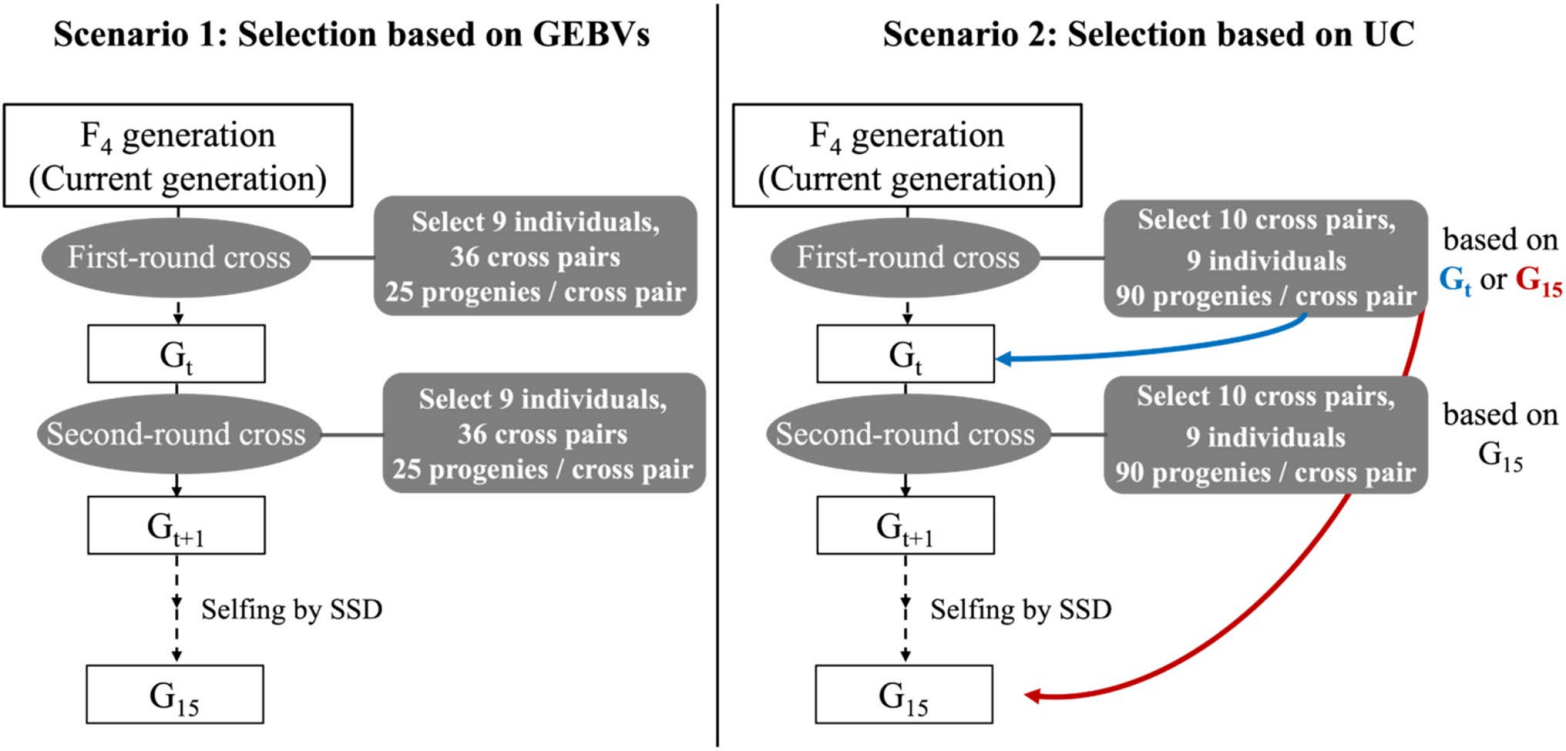
Breeding program used in this study. SSD, single-seed descent. In Scenario 2, two approaches were used for selecting cross pairs in the first-round cross: based on the genotypic values of either the G_t_​ generation or the G_15_​ generation. In the second round of cross, cross pairs were selected based on the genotypic values of the G_15_​ generation

Stochastic simulations of breeding programs were performed to evaluate the effectiveness of interpopulation crosses for multi-trait improvement. An outline of the breeding program is shown in Fig. 2. Two scenarios were investigated in this study. In both scenarios, a GP model was constructed for each population in the F_4_ generation (initial generation used in this study), and the first round of selection and crossing was performed in the same generation. The second round of selection and crossing is implemented in generation G_t_﻿ ($t=1, 2, 3, 4$), which refers to the $t$ ^th^ generation after the initial cross, i.e., the first round of selection and crossing. In generations without selection or crossing, all individuals underwent self-pollination, and the next generation was obtained through single-seed descent (SSD). To enable using different marker effects for each population, a simulation code was designed to distinguish the origin of alleles from each population. The R code used for the genome simulation is available in the Data Availability section. A brief description of the method developed to track alleles originating from different populations is provided in Supplementary File 4. All simulations were performed using R version 4.4.1.

In Scenario 1, high-performing individuals with high genotypic values were selected for crossing. In Scenario 2, cross pairs that could generate superior progeny were selected by predicting the genotypic value of their progeny. The selection of cross pairs in Scenario 2 was based on the predicted genotypic value, calculated using the UC, of the generation used for the second-time cross (G_t_) or the final generation (G_15_). For scenarios where the first-time cross selection was based on G_15_, we did not consider selection and crossing at the second-time cross (G_t_). Instead, the UC was calculated directly for G_15_ and used to identify cross pairs for the first-time cross. The breeding program was designed to span 15 generations to ensure genetic fixation. The details of Scenarios 1 and 2 are described in the following section. The simulations were repeated 50 times for each scenario, and the genetic gains were averaged across repetitions to compare the different scenarios.

### Scenario 1: individual-based selection

In Scenario 1, nine individuals (five from the S827 population and four from the S840 population) were selected from the F_4_ generation based on the highest selection index, which was defined as the sum of the genotypic values of perillaldehyde and rosmarinic acid contents. The index for individual $i$ is calculated as follows:

3 $${u}_{i}={\sum }_{m=1}^{2}{u}_{im}={\sum }_{m=1}^{2}{\sum }_{p=1}^{2}{\mathbf{x}}_{ip}{{\varvec{\upbeta}}}_{pm}$$

where ${u}_{im}$ represents the scaled genotypic values of the individual $i$ ($i=1, \dots , 595$) for the $m$ ^th^ trait; ${\mathbf{x}}_{ip}$ is an $1\times L$ vector corresponding to the marker genotype of the individual $i$ belonging to the $p$ ^th^ population; ${{\varvec{\upbeta}}}_{pm}$ denotes the estimated marker effects of the $m$ ^th^ trait and $p$ ^th^ population; and $L$ is the total number of markers ($L=\text{1,951}$). Nine selected individuals were crossed in all possible combinations without selfing, resulting in 36 combinations. A total of 25 progenies produced from each cross were collected, with a population size of 900. In the G_t_ generation, selection was performed in the same manner as for the first cross. Nine individuals with the highest selection indices were selected from entire pool of 900 individuals in the G_t_ generation and crossed in all possible combinations to generate for the next generation.

### Scenario 2: cross-pair-based selection

In Scenario 2, 10 cross pairs, including nine individuals from the F_4_ generation, were selected based on UC. UC was used to evaluate the potential of a cross to generate superior progeny and was calculated as follows:

4 $${UC}_{k}={\mu }_{k}+i\sqrt{{\sigma }_{k}^{2}}$$

where ${\mu }_{k}$ is the mean genotypic value of cross $k$, $i$ is the selection intensity (set to 1.96 in this study), and ${\sigma }_{k}^{2}$ is the genetic variance of the progeny generated from cross $k$. The selection intensity was set to 1.96 because, in this study, we assumed a small-scale breeding program, and this value reflects a realistic number of individuals that can be crossed and selected in actual perilla breeding. The genetic variance, ${\sigma }_{k}^{2}$, can be calculated analytically for any generation of inbred lines (Lehermeier et al. 2017). For this study, we combined the methodologies outlined by Allier et al. (2019) and Danguy des Déserts et al. (2023) to apply the calculation of ${\sigma }_{k}^{2}$ to interpopulation crosses, assuming different genetic effects among populations. ${\sigma }_{k}^{2}$ was computed as follows:

5 $${\sigma }_{km}^{2}={{\sigma }_{km}^{2}}^{(12)}+{{\sigma }_{km}^{2}}^{\left(34\right)}+{{\sigma }_{km}^{2}}^{(13)}+{{\sigma }_{km}^{2}}^{\left(14\right)}+{{\sigma }_{km}^{2}}^{\left(23\right)}+{{\sigma }_{km}^{2}}^{\left(24\right)}$$

where alleles 1 and 2 are derived from parent 1 and alleles 3 and 4 are derived from parent 2. ${{\sigma }_{km}^{2}}^{\left(12\right)}$ represents the genetic variance between alleles 1 and 2 derived from parent 1 for the $m$ ^th^ trait, and ${{\sigma }_{km}^{2}}^{\left(34\right)}$ represents the genetic variance between alleles 3 and 4 derived from parent 2 for the $m$ ^th^ trait. The terms ${{\sigma }_{km}^{2}}^{\left(13\right)}$, ${{\sigma }_{km}^{2}}^{\left(14\right)}$, ${{\sigma }_{km}^{2}}^{\left(23\right)}$, and ${{\sigma }_{km}^{2}}^{\left(24\right)}$ represent the genetic variances between two alleles derived from different parents. The genetic variance ${{\sigma }_{km}^{2}}^{\left(12\right)}$ can be computed as follows:

6 $${{\sigma }_{km}^{2}}^{\left(12\right)}={\left\{\left({\mathbf{x}}_{1}^{\left(1\right)}\circ {{\varvec{\upbeta}}}_{1m}+{\mathbf{x}}_{2}^{(1)}\circ {{\varvec{\upbeta}}}_{2m}\right)-\left({\mathbf{x}}_{1}^{\left(2\right)}\circ {{\varvec{\upbeta}}}_{1m}+{\mathbf{x}}_{2}^{(2)}\circ {{\varvec{\upbeta}}}_{2m}\right)\right\}}^{\text{T}}{\mathbf{D}}^{(12)}\left\{\left({\mathbf{x}}_{1}^{\left(1\right)}\circ {{\varvec{\upbeta}}}_{1m}+{\mathbf{x}}_{2}^{(1)}\circ {{\varvec{\upbeta}}}_{2m}\right)-\left({\mathbf{x}}_{1}^{\left(2\right)}\circ {{\varvec{\upbeta}}}_{1m}+{\mathbf{x}}_{2}^{(2)}\circ {{\varvec{\upbeta}}}_{2m}\right)\right\}$$

where ${\mathbf{x}}_{1}^{\left(1\right)}$ and ${\mathbf{x}}_{1}^{ \left(2\right)}$ are $L\times 1$ vectors representing the haplotype scores of alleles 1 and 2 (coded as 0 or 1), respectively, for parent 1 from population 1; ${{\varvec{\upbeta}}}_{1m}$ is an $L\times 1$ vector of marker effects from population 1 for trait $m$; ${\mathbf{x}}_{2}^{\left(1\right)}$ and ${\mathbf{x}}_{2}^{ \left(2\right)}$ are $L\times 1$ vectors representing the haplotype scores of alleles 1 and 2, respectively, for parent 1 from population 2; ${{\varvec{\upbeta}}}_{2m}$ is an L×1 vector of marker effects from population 2 for trait $m; {\mathbf{x}}_{1}^{\left(1\right)}\circ {{\varvec{\upbeta}}}_{1m}$ represents the Hadamard product of two vectors; and ${\mathbf{D}}^{\left(12\right)}$ is an $L\times L$ variance–covariance matrix of linkage disequilibrium between alleles 1 and 2 at different loci, which is common in all crosses. The genetic variance ${{\sigma }_{km}^{2}}^{\left(34\right)}$ between the two alleles from parent 2 is computed similarly. Moreover, the genetic variance between alleles derived from different parents, such as ${{\sigma }_{km}^{2}}^{\left(13\right)}$, ${{\sigma }_{km}^{2}}^{\left(14\right)}$, ${{\sigma }_{km}^{2}}^{\left(23\right)}$, and ${{\sigma }_{km}^{2}}^{\left(24\right)}$, can also be calculated. For example, ${{\sigma }_{km}^{2}}^{\left(13\right)}$ can be calculated as follows:

7 $${{\sigma }_{km}^{2}}^{\left(13\right)}={\left\{\left({\mathbf{x}}_{1}^{\left(1\right)}\circ {{\varvec{\upbeta}}}_{1m}+{\mathbf{x}}_{2}^{(1)}\circ {{\varvec{\upbeta}}}_{2m}\right)-\left({\mathbf{x}}_{1}^{\left(3\right)}\circ {{\varvec{\upbeta}}}_{1m}+{\mathbf{x}}_{2}^{(3)}\circ {{\varvec{\upbeta}}}_{2m}\right)\right\}}^{\text{T}}{\mathbf{D}}^{\left(13\right)}\left\{\left({\mathbf{x}}_{1}^{\left(1\right)}\circ {{\varvec{\upbeta}}}_{1m}+{\mathbf{x}}_{2}^{(1)}\circ {{\varvec{\upbeta}}}_{2m}\right)-\left({\mathbf{x}}_{1}^{\left(3\right)}\circ {{\varvec{\upbeta}}}_{1m}+{\mathbf{x}}_{2}^{(3)}\circ {{\varvec{\upbeta}}}_{2m}\right)\right\}$$

where ${\mathbf{x}}_{1}^{\left(3\right)}$ and ${\mathbf{x}}_{2}^{(3)}$ represent the $L\times 1$ vectors of the haplotype score of the allele 3 for parent 2 from populations 1 and 2, respectively, and ${{\varvec{\upbeta}}}_{1m}$ and ${{\varvec{\upbeta}}}_{2m}$ are the $L\times 1$ vectors of marker effects from populations 1 and 2, respectively, for trait $m$. The linkage disequilibrium matrix $\mathbf{D}$ can be computed based on the recombination rate, as follows:

8 $${\mathbf{D}}^{\left(12\right)}={\mathbf{D}}^{\left(34\right)}=0.25\left(1-{\mathbf{C}}^{g} \right)\left(1-2{\mathbf{C}}^{1}\right)$$

9 $${\mathbf{D}}^{\left(13\right)}={\mathbf{D}}^{\left(14\right)}={\mathbf{D}}^{\left(23\right)}={\mathbf{D}}^{\left(24\right)}=0.25\left(1-2{\mathbf{C}}^{g}-\left(0.5{\left(1-2{\mathbf{C}}^{1}\right)}^{g-1}\right)\right)$$

where ${\mathbf{C}}^{g}=\frac{2{\mathbf{C}}^{1}\left(1-{0.5}^{g}{\left(1-2{\mathbf{C}}^{1}\right)}^{g}\right)}{1+2{\mathbf{C}}^{1}}$ is an $L\times L$ matrix of recombination rates in the $g$ ^th^ generation, and ${\mathbf{C}}^{1}$ is an $L\times L$ matrix of recombination rates between markers. Note that when $g=1$, ${\mathbf{C}}^{g}$​ corresponds to ${\mathbf{C}}^{1}$​. The $g$ ^th^ generation here implies the $g-1$ selfing generation after crossing (Fig. S3). The diagonal elements of $\mathbf{D}$ are 0.25 when $g=1$ and ${{\sigma }_{km}^{2}}^{\left(13\right)}={{\sigma }_{km}^{2}}^{\left(14\right)}={{\sigma }_{km}^{2}}^{\left(23\right)}={{\sigma }_{km}^{2}}^{\left(24\right)}=0$. Among Eqs. 5, 6, 7, 8, and 9, Eqs. 5, 8, and 9 are based on the study by Allier et al. (2019), whereas Eqs. 6 and 7 are derived from the Supplementary Files of Danguy des Déserts et al. (2023). In Eqs. 6 and 7, we modified the equations from Danguy des Déserts et al. (2023) to allow the marker effects to differ depending on the population (i.e., the population to which the crossed parents belong). We present a worked toy example to facilitate elucidating Eqs. 6 and 7 in Supplementary File 2.

In this scenario, cross pairs with the highest selection index, defined as the sum of the UC of the two traits (${\sum }_{m=1}^{2}{UC}_{km}$), were selected. Here, ${\mu }_{km}$, representing the mean genotypic value of cross $k$ for trait $m$, was scaled using the mean and standard deviation of genotypic values from the F_4_ generation. Similarly, ${\sigma }_{km}^{2}$, representing the genetic variance of progeny generated from cross $k$ for trait $m$, was scaled only using the standard deviation of genotypic value from the F_4_ generation. After selecting the cross pairs based on UC, 90 progenies produced from each cross were collected to maintain a population of 900 individuals. In the G_t_ generation, cross pairs were selected in the same manner as the first cross, meaning that 10 cross pairs were chosen from the entire 900 individuals in the G_t_ generation. However, in contrast to the first cross, to reduce the computation time, UC was calculated for all possible combinations of the 450 individuals with the highest selection indices, and 10 cross pairs with the highest UC were subsequently selected to generate the progeny.

In the first-round crosses, 16 pairs in Scenario 1 were intra-population crosses, and in some cases, intra-population crosses were included in Scenario 2. However, from the second round onward, alleles from S827 and S840 were mixed. By the final generation (G_15_), all individuals in both scenarios were confirmed to possess alleles from both S827 and S840 across all simulation runs.

### Comparative analyses

**Fig. 3:**
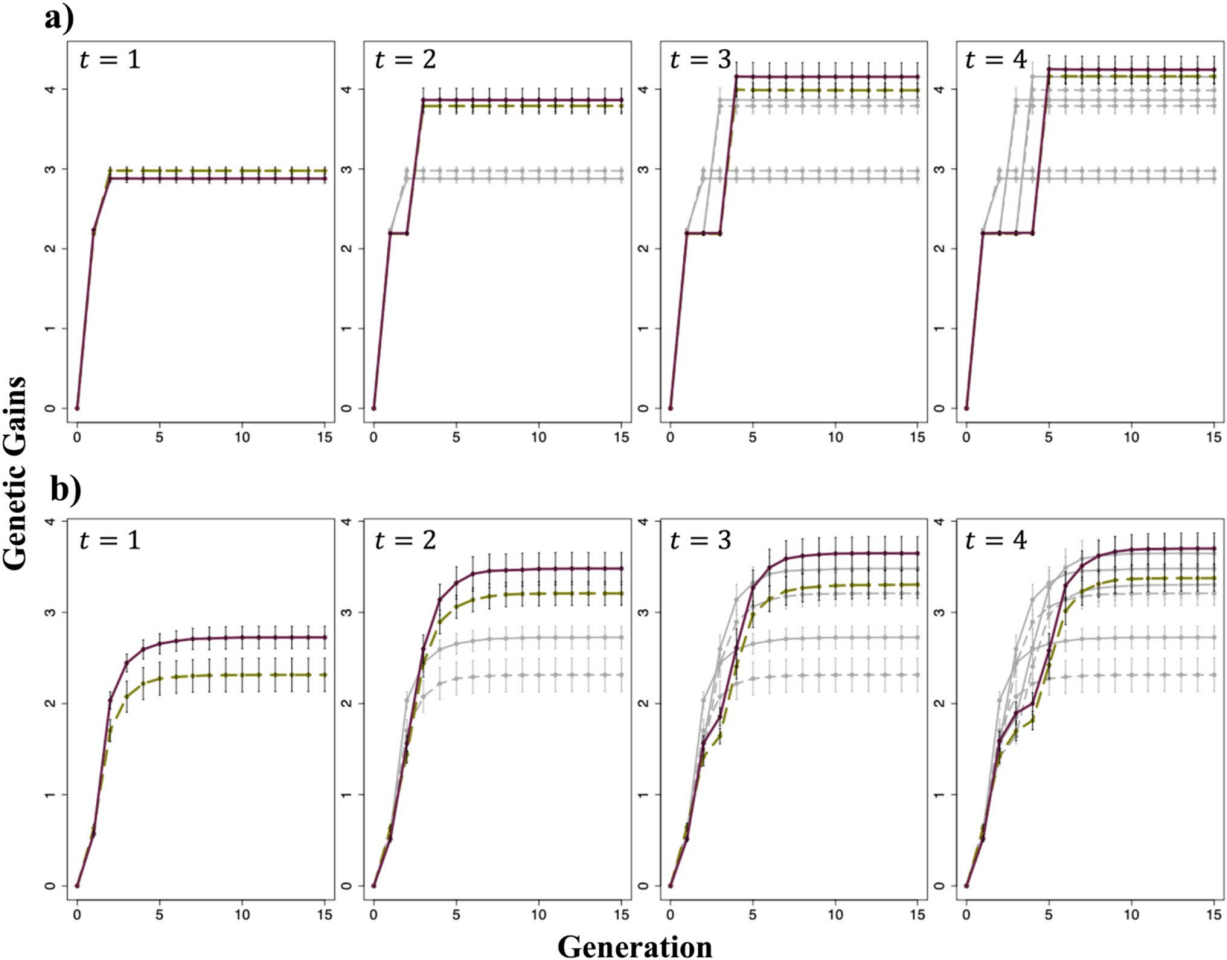
Changes in the mean genetic gains of the entire population and top 1% of individuals evaluated for selection index. Genetic gains were calculated using Eq. (10). **a** Changes in the mean genetic gains of the entire population. **b** Change in the mean genetic gains of the top 1% of individuals. *t* represents the generation at which the second-round cross was made. Green dashed line: Scenario 1; Purple solid line: Scenario 2; Gray dashed line: when the second-round crosses were performed at earlier generations than those indicated as *t* for Scenario 1; Gray solid line: when the second-round crosses were performed at earlier generations than those indicated as *t* for Scenario 2. In Scenario 2, the cross pairs for the first-round cross were selected based on the estimated genotypic values of the generation at which the second-round cross was performed. The error bars represent the mean ± standard deviation of the genetic gain obtained from 50 simulations

**Fig. 4:**
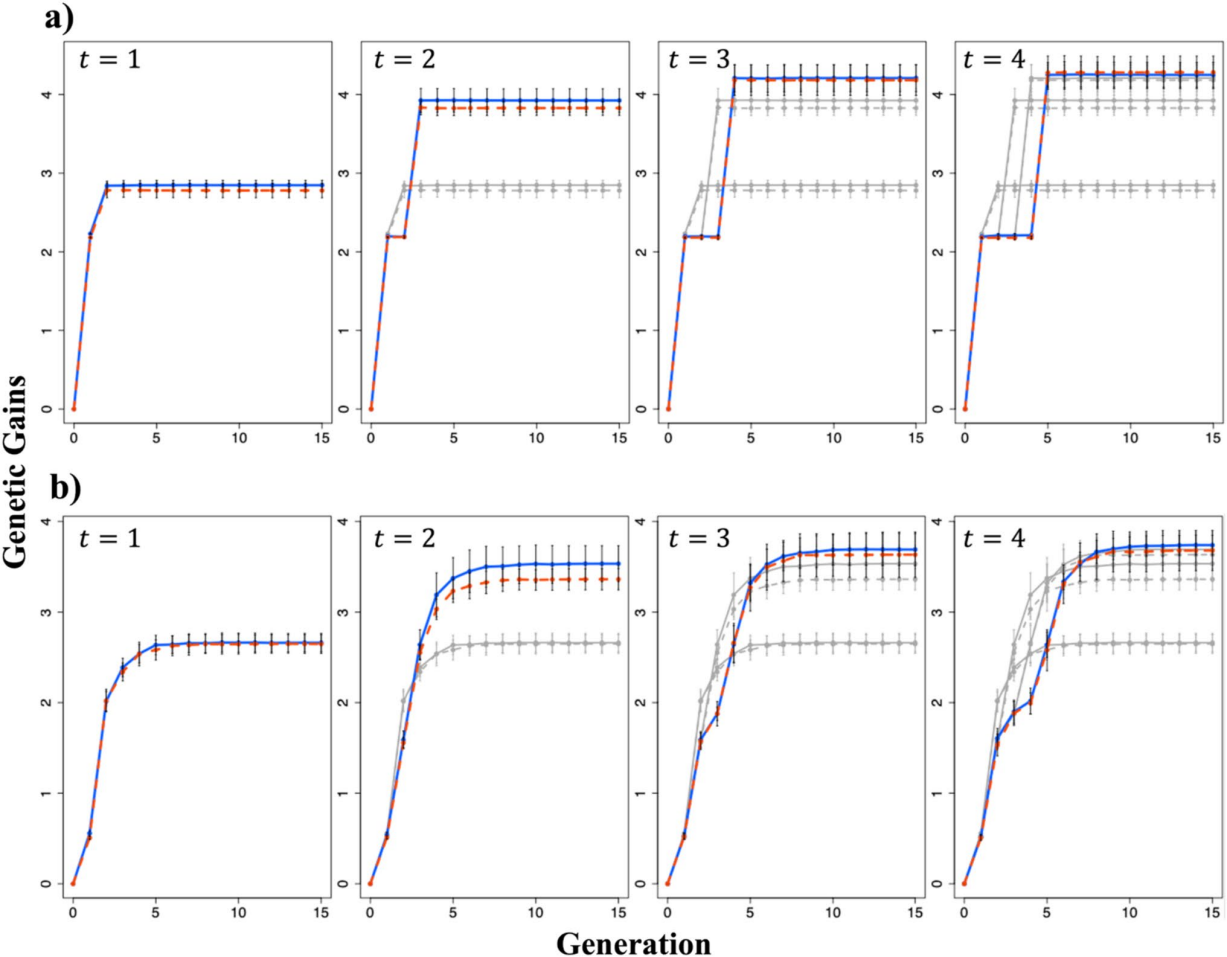
Changes in the mean genetic gains of the entire population and top 1% of individuals when the cross pairs for the first-round cross were selected based on the predicted genotypic values of the different generations of progenies in Scenario 2. Genetic gains were calculated using Eq. (10). **a** Changes in the mean genetic gains of the entire population. **b** Changes in the mean genetic gains of the top 1% of individuals. *t* represents the generation at which the second-round cross was made. Red dashed line: when the cross pairs for the first-round cross were selected based on the predicted genotypic value of the final generation; Blue solid line: when the cross pairs for the first-round cross were selected based on the predicted genotypic value of the generation used for the second-round cross; Gray lines: when the second-round crosses were performed at earlier generations than those indicated as *t*, and the line types are consistent with the colored lines. The error bars represent the mean ± standard deviation of the genetic gains obtained from 50 simulations

We performed 50 breeding simulations for both scenarios. For comparison, we computed the mean genetic gains of the entire population and the top 1% of individuals based on the data of the simulated individuals in each generation as follows:

10 $$G\left(t\right)=\frac{\stackrel{-}{u\left(t\right)}-\stackrel{-}{u(0)}}{\sqrt{{\sigma }_{g}^{2}}}$$

where in Figs. 3a and 4(a), $\stackrel{-}{u\left(t\right)}$ and $\stackrel{-}{u(0)}$ represent the mean selection index in generation G_t_ ($t=1, 2, \dots , 15$) and the initial F_4_ population, respectively, for the entire population. Figures 3(b) and 4(b) represent the mean selection index for the top 1% of individuals in generation G_t_ and in the initial F_4_ population, respectively. In Fig. 7(a), $\stackrel{-}{u\left(t\right)}$ and $\stackrel{-}{u(0)}$ represent the mean genotypic values for each trait in generation G_t_ and the initial F_4_ population, respectively, for the top 1% of individuals. In all cases, ${\sigma }_{g}^{2}$ is the genetic variance of individuals in the initial F_4_ population.

We also computed the genetic variance of each generation as follows:

11 $${\sigma }^{2}\left(t\right)=\frac{\text{var}\left(u\left(t\right)\right)}{{\sigma }_{g}^{2}}$$

where $\text{var}\left(u\left(t\right)\right)$ is the genetic variance of individuals in the generation G_t_, and ${\sigma }_{g}^{2}$ is the genetic variance of individuals in the initial F_4_ population.

**Fig. 5:**
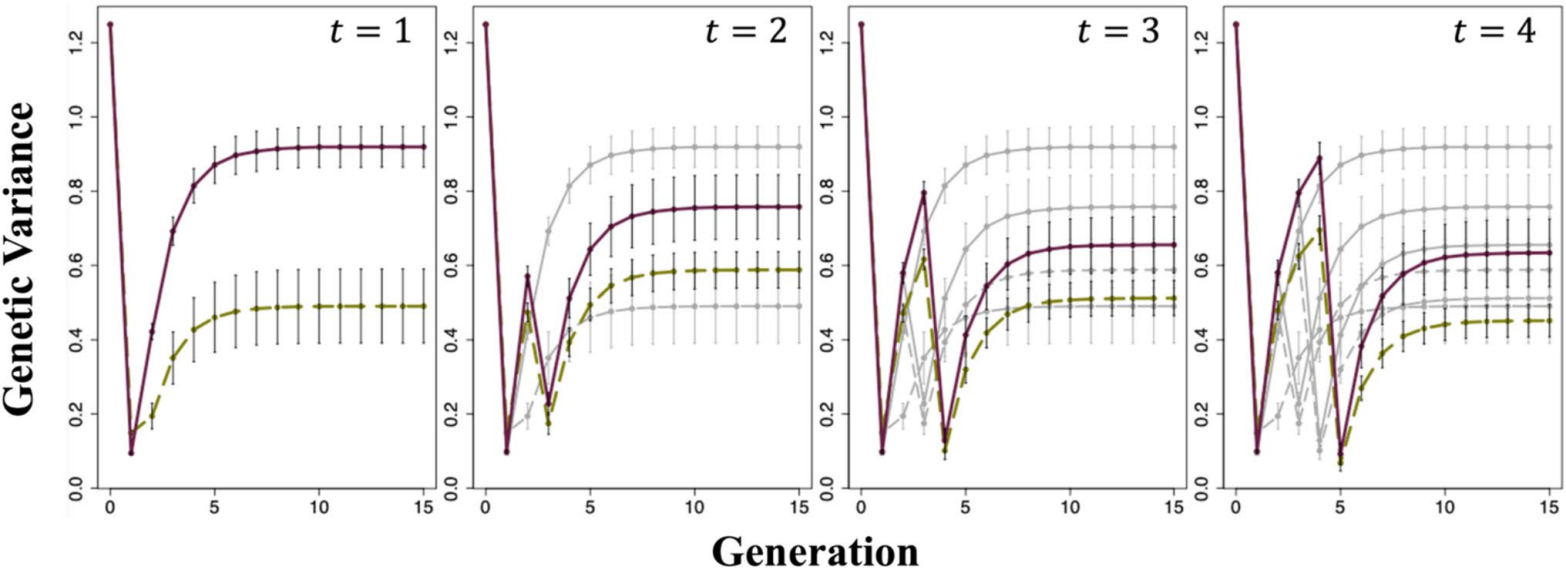
Changes in the genetic variance of the selection index. The genetic variance was calculated using Eq. (11). *t* represents the generation at which the second-round cross was made. Green dashed line: Scenario 1; Purple solid line: Scenario 2; Gray dashed line: when the second-round crosses were performed at earlier generations than those indicated as *t* for Scenario 1; Gray solid line: when the second-round crosses were performed at earlier generations than those indicated as *t* for Scenario 2. In Scenario 2, the cross pairs for the first-round cross were selected based on the estimated genotypic values of the generation at which the second-round cross was performed. The error bars represent the mean ± standard deviation of the genetic gain obtained from 50 simulations

**Fig. 6:**
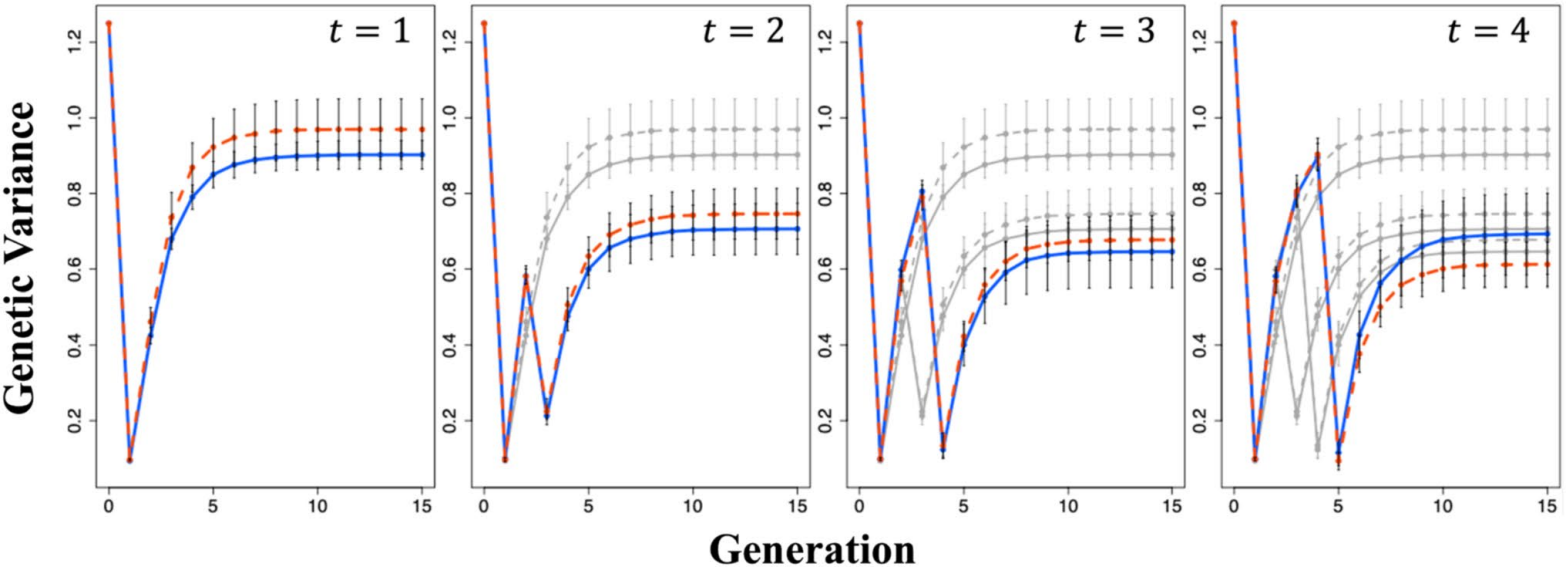
Changes in genetic variance when the selection of the cross pairs for the first-round cross was based on the predicted genotypic values of the different generations of progeny in Scenario 2. The genetic variance was calculated using Eq. (11). *t* represents the generation at which the second-round cross was made. Red dashed line: when the cross pairs for the first-round cross were selected based on the predicted genotypic values of the final generation; Blue solid line: when the cross pairs for the first-round cross were selected based on the predicted genotypic values of the generation used for the second-round cross; Gray lines: when the second-round crosses were performed at earlier generations than those indicated as *t*, and the line types are consistent with the colored lines. The error bars represent the mean ± standard deviation of the genetic gains obtained from 50 simulations

**Fig. 7:**
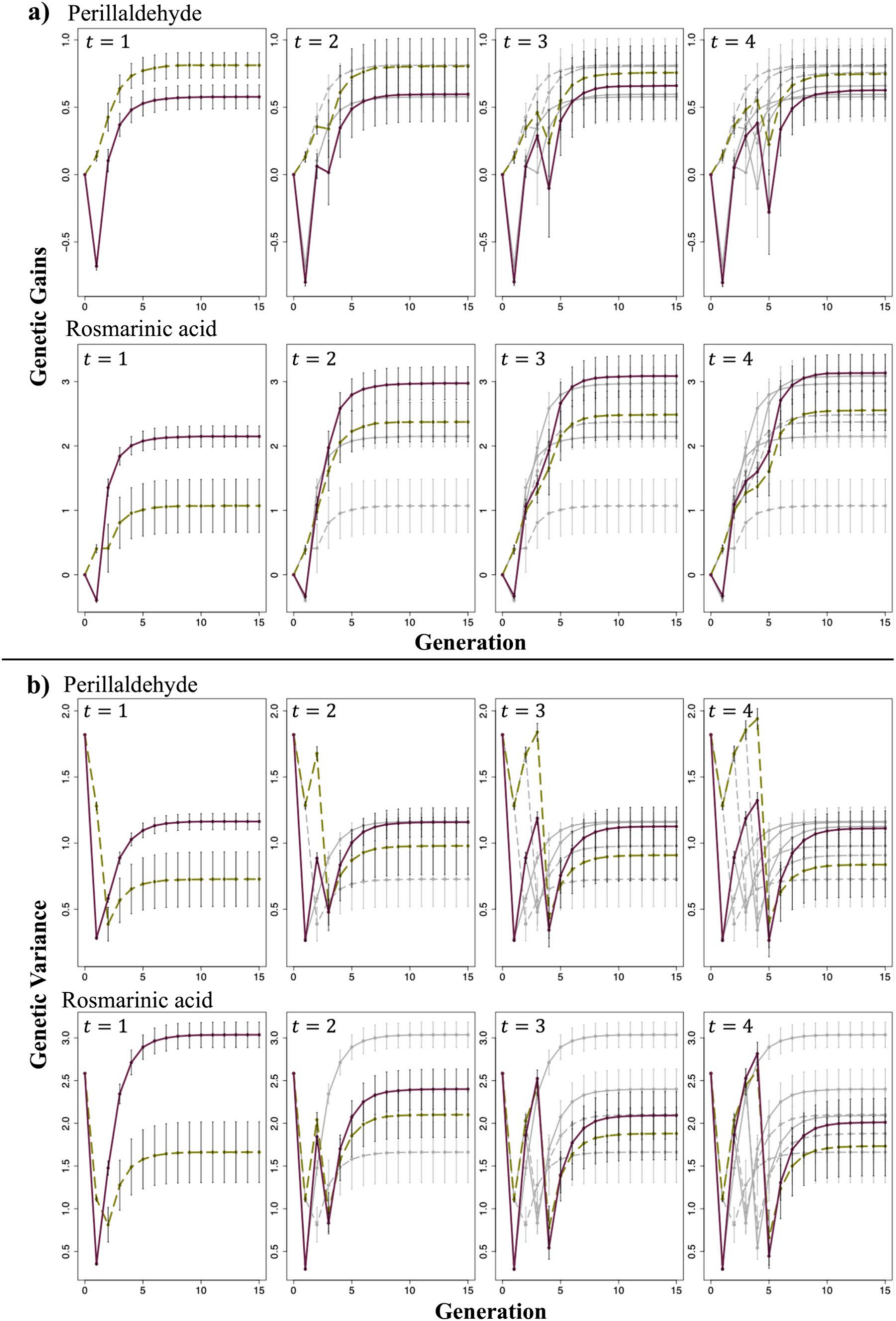
Changes in the mean genetic gains and genetic variance of the top 1% of individuals evaluated for each trait. Genetic gains and genetic variance were calculated using Eqs. (10) and (11), respectively. **a** Changes in the mean genetic gains of the top 1% of individuals. **b** Changes in the genetic variance of the top 1% of individuals. *t* represents the generation at which the second-round cross was made. Green dashed line: Scenario 1; Purple solid line: Scenario 2; Gray dashed line: when the second-round crosses were performed at earlier generations than those indicated as *t* for Scenario 1; Gray solid line: when the second-round crosses were performed at earlier generations than those indicated as *t* for Scenario 2. In Scenario 2, the cross pairs for the first-round cross were selected based on the genotypic values of the generation at which the second-round cross was performed. The error bars represent the mean ± standard deviation of the genetic gain or genetic variance obtained from 50 simulations

In the Results section, the error bars shown in Figs. 3, 4, 5, 6 and 7 were created using the mean ± standard deviation of $G\left(t\right)$ or ${\sigma }^{2}\left(t\right)$ from 50 simulations.

## Results

### ‘SekihoS8’ reference genome construction

We obtained 2,011,482 HiFi reads with a length of 36.4 Gb, which were assembled using hifiasm v0.16.1 to construct a primary assembly and two haplotype assemblies (Hap1 and Hap2). After removing the mitochondrial and chloroplast genomes, 108, 121, and 106 assembled sequences were obtained for the primary, Hap1, and Hap2 assemblies, respectively (Table S1). The total lengths of the assemblies ranged from 1,231 Mb to 1,259 Mb, with N50 values ranging from 55.4 Mb to 56.0 Mb. When aligned to the ‘Hoko-3’ genome, all contigs from these assemblies were successfully mapped to 20 chromosomal sequences, with no major structural variations detected (Fig. S4). The results of the Smudgeplot analysis suggested that ‘SekihoS8’ is a potential allotetraploid (Fig. S5). In addition, telomere sequences were detected in the terminal regions of most chromosome-scale scaffold sequences (Fig. S6a–c). The constructed genomes were designated as Pfru_SekihoUp_1.0 (primary), Pfru_SekihoH1_1.0 (hap1), and Pfru_SekihoH2_1.0 (hap2). BUSCO analysis revealed that the percentage of complete genes in all three genome assemblies was 99.4%, indicating high completeness and coverage of known genes. Gene prediction using a helixer identified 65,161 genes in the primary assembly: 65,078 in Hap1 and 62,784 in Hap2 (Table S2). The results of the BUSCO analysis further confirm its high accuracy, with complete BUSCO scores ranging from 98.7 to 99.1%. Duplicate complete BUSCOs showed proportions of 89.0% for Hap2 and 99.1% for the primary and Hap1 assemblies, indicating high levels of genome duplication.

### Binomial test for scenario comparison

The frequency with which Scenario 2 outperformed Scenario 1 in terms of genetic gains for the selection index and individual trait (population mean, top 1% individual mean, and genetic variance) in the final generation (G_15_) across all simulations is presented in Table S3. To assess the superiority of Scenario 2, we conducted a binomial test under the null hypothesis that both scenarios had equal probability of outperforming in each simulation. Based on selection index, Scenario 2 significantly outperformed Scenario 1 in terms of the mean genetic gains across the entire population, the mean genetic gains of the top 1% of individuals, and genetic variance, with the exception of the mean genetic gains of the entire population when t = 1. When evaluating individual traits, Scenario 2 did not demonstrate a consistent advantage for perillaldehyde regarding either population-wide mean genetic gains or gains among the top 1% of individuals. However, for rosmarinic acid, Scenario 2 consistently yielded superior gains across all metrics.

### Changes in genetic gains for selection index

The changes in the mean genetic gains of the entire population and those of the top 1% of individuals from the initial population (F_4_ generation) to the final generation (G_15_) are shown in Fig. 3. Regarding the mean genetic gains of the entire population, Scenario 2 showed greater improvement than that in Scenario 1 in almost all cases, except for when a second-round cross was performed between the individuals of the G_1_ generation. In all cases, Scenario 2 considerably outperformed Scenario 1 in the mean genetic gains of the top 1% of individuals. The mean genetic gains of the entire population and the top 1% of individuals were higher when the second-round cross was performed in a later generation, although the extent of improvement diminished as the generations progressed. The changes in genetic gains when the first-round cross pairs in Scenario 2 were selected based on the genotypic values of the different generations of progeny are shown in Fig. 4. The mean genetic gain of the entire population and that of the top 1% of individuals were slightly higher when pairs for the first-round cross were selected based on the predicted genotypic value of the generation used for the second-round cross rather than those of individuals in the cross pairs.

### Changes in genetic variance

The changes in genetic variance when the first-round cross pairs were selected based on the predicted genotypic values of the generation used for the second-round cross in Scenario 2 are shown in Fig. 5. Scenario 2 maintained a higher genetic variance in all cases. The timing of the second-round cross affected genetic variance, with later crosses resulting in reduced genetic variance in the final generation (G_15_) but increased genetic variance in the generation at which the second-round cross was made (*G*_*t*_, where $t=\text{1,2},\text{3,4}$).

Moreover, the changes in genetic variance when the first-round cross pairs were selected based on the genotypic values of the different generations of progeny in Scenario 2 are shown in Fig. 6. The genetic variance was nearly the same in the generation used for the second-round cross but higher in G_15_ when the cross pairs for the first-round cross were selected based on the predicted genotypic values of G_15_ rather than those of the generation used for the second-round cross. However, the opposite trend was observed when a second-round cross was performed in the G_1_ generation.

### Changes in genetic gains and genetic variance for each analyzed trait

The changes in the genetic gains of the top 1% of individuals and the genetic variance for each trait, namely perillaldehyde and rosmarinic acid contents, are shown in Fig. 7. Regarding the mean genetic gains of the top 1% of individuals, Scenarios 1 and 2 resulted in enhanced genetic improvements in perillaldehyde and rosmarinic acid contents, respectively. Regarding genetic variance, Scenario 2 exhibited higher variance for both perillaldehyde and rosmarinic acid content than Scenario 1 in the final generation (G_15_) and lower variance in the generation used for the second-round cross. Genetic variance in the final generation and the generation used for the second-round crossing varied depending on the timing of the second-round crossing. The descending order of genetic variance in the final generation for scenarios 1 and 2 was G_2_ > G_3_ > G_4_ > G_1_ and G_1_ > G_2_ > G_3_ > G_4_, respectively. Similarly, the descending order of genetic variance in the generation used for the second-round cross for scenarios 1 and 2 was G_4_ > G_3_ > G_2_ > G_1_.

## Discussion

In the present study, we evaluated the effectiveness of using UC to select cross pairs for interpopulation crosses aimed at simultaneously improving multiple traits. Additionally, to propose specific guidelines for interpopulation crosses in the context of GS, we examined the optimal timing for mating. When assessing genetic gains using the selection index, Scenario 2, which employed UC, showed no noticeable difference in the mean genetic gains of the entire population compared with conventional selection based on genotypic value; however, it considerably outperformed conventional selection in terms of the mean genetic gains of the top 1% of individuals (Fig. 3). This result can be attributed to Scenario 2, which maintained a higher genetic variance across generations. Previous studies have also indicated that maintaining high genetic variance plays a crucial role in the genetic improvement of a population (Jannink et al. 2010). In Scenario 1, individuals were selected based on the mean genotypic value, which likely allowed crosses between genetically similar individuals. In contrast, in Scenario 2, cross pairs were selected based on UC, considering both mean genotypic values and genetic variance, thereby ensuring improved compatibility between parents. Moreover, the greatest genetic gains were observed when the second-round cross was performed in G_4_, likely because of the higher genetic variance maintained in that generation than in the other generations. Figure S7 shows changes in genetic variance when inbreeding via SSD was repeated after a single cross. Genetic variance initially increased because of segregation from the cross but gradually plateaued as the population became genetically fixed. Performing crosses over generations with high genetic variance enables the selection of superior individuals, leading to enhanced genetic improvement. In extreme cases, performing crosses when the population is genetically fixed results in the maximum genetic improvement. However, considering the balance between the costs of accelerating generations and the degree of improvement achieved through crosses, the optimal timing for crossing is likely around G_3_ and G_4_ when the increase in genetic variance starts to plateau.

Moreover, when focusing on improvement in each trait, Scenario 1, wherein the genotypic value was used for selection, demonstrated superior performance to Scenario 2 regarding the improvement in perillaldehyde content. In contrast, Scenario 2, wherein UC was utilized to select cross pairs, showed a greater improvement in rosmarinic acid content than that in Scenario 1 (Fig. 7). When examining the final genetic gains for each trait, perillaldehyde content exhibited minimal improvement compared with that in the initial generation; however, substantial improvements were observed in rosmarinic acid content. This result suggests that Scenario 2, which effectively facilitated an improvement in rosmarinic acid content, outperformed Scenario 1 in terms of improvement in the selection index. When comparing the genotypic values of ideal individuals, i.e., those possessing favorable alleles at all markers, with the maximum genotypic values of the initial population, perillaldehyde content achieved a multiple of 3.78, whereas rosmarinic acid content achieved a multiple of 5.37 (data not shown). According to Kinoshita et al. (2023), perillaldehyde content is regulated by a limited number of major genes with high effects, whereas rosmarinic acid was controlled by genes with relatively small effects. The limited improvement in perillaldehyde levels can be attributed to the accumulation of favorable alleles in major genes in the initial generation of superior individuals. Conversely, improving rosmarinic acid content required the accumulation of favorable alleles across many markers, indicating an enhanced potential for improvement. Allier et al. (2019) suggested that allele pyramiding is more suitable for traits controlled by a few major QTLs than those controlled by many minor QTLs. In addition, analyzing the differences in genetic gain between the two traits requires consideration of their genetic architecture and heritability, and how these factors influence to UC. The relationship between heritability and the prediction accuracy of UC has been examined in previous studies (Rembe et al. 2022; Wang et al. 2024). Though heritability in these studies was estimated using phenotypic rather than genomic data, the findings consistently show that lower heritability diminishes UC prediction accuracy. In our study, estimated marker effects were treated as true values, thereby not accounting for potential reduction in UC prediction accuracy due to low genomic heritability. However, Kinoshita et al. (2023) reported lower genomic heritability of rosmarinic acid compared to perillaldehyde, suggesting that the genetic improvement observed in our simulations may be attenuated in practical breeding applications. Moreover, Wang et al. (2024) demonstrated that for traits controlled by numerous genes, accuracy in estimating progeny genetic variance tends to decrease, while UC prediction accuracy improves. This indicates that for highly polygenic traits, the mean component dominates variance in UC prediction. Therefore, when applying UC in practical breeding programs, careful consideration of the target trait’s genetic architecture is essential.

In Scenario 2, selecting cross pairs from the initial population based on the predicted genotypic value of the generation used for the second-round cross rather than that of G_15_ resulted in slightly greater genetic gains (Fig. 4), likely because UC introduces a slight prediction bias when selection is applied (Allier et al. 2019). Therefore, when the predicted genotypic value of the final generation is used as the basis, the selection of cross pairs based on the predicted genotypic value of the generation used for the second-round cross may cause a deviation in the distribution of progeny genotypic values in the final generation compared to that in the initial prediction.

In this study, the estimated marker effects were used as true marker effects in breeding simulation, and no updates to the GP model were performed. However, it has been asserted that the prediction accuracy of the GP model is directly related to the effectiveness of selection based on UC (Müller et al. 2018). Although the advantage of selecting cross pairs using UC with model updates has been demonstrated in a simulation study assuming recurrent selection (Sakurai et al. 2024), future research should consider the prediction accuracy of GP and heritability.

Additionally, in this study, the selection intensity ($i$) used to calculate UC was set to 1.96, based on practical experience from our ongoing perilla (*Perilla frutescens*) breeding program. However, the actual selection intensity observed in the simulations differed from this fixed value and varied depending on whether UC was calculated based on the G_t_ or G_15_ generation. When calculated based on the G_15_ generation, the final genetic gain was evaluated as the average of the top 1% of individuals, meaning that nine out of 900 individuals were selected. In contrast, when UC was calculated based on the G_t_ generation, ten cross pairs were selected, and the number of individuals involved in these crosses varied among simulation replicates, resulting in replicate-specific selection intensities. If UC had been calculated using the actual selection intensity corresponding to each stage in the simulations (i.e., the second-round crosses or the G_15_ generation), the final genetic gain might have been affected. Although further research is needed to fully understand the relationship between selection intensity in UC calculations and the resulting genetic gain, we conducted a simple exploratory analysis to assess the robustness of UC with respect to varying selection intensities.

Using data from this study, we calculated UC using Eq. 4 with $k=$ 15 and setting $i=$ 1.64, 1.96, and 2.58 (corresponding to selection of the top 10%, 5%, and 1% of the population, respectively). Although the rankings of the top ten crosses differed slightly between $i=$ 1.64 and $i=$ 1.96, the selected cross pairs were completely consistent. When $i=$ 2.58 was used, eight of the top 10 crosses overlapped with those selected at lower intensities (data not shown). These results suggest that changing the selection intensity primarily alters the weights assigned to the mean and variance in the UC calculation. Because the contribution of variance tended to be smaller than that of the mean, the set of selected crosses remained relatively stable across a range of selection intensities. Nevertheless, further investigation is warranted to better understand the impact of selection intensity on UC performance, particularly for practical applications in breeding programs.

Such studies will help advance our understanding of the effectiveness of interpopulation crosses in the context of GS.

The interpopulation cross is a breeding method used empirically when improving multiple traits within a single biparental population is challenging. In this study, we used two red perilla biparental populations as the material, which required the simultaneous improvement of multiple medicinal compounds. However, this method can only be applied to self-pollinating crops with more than two biparental populations and is particularly effective for crops that have yet to achieve considerable improvement in genetic traits through breeding programs, such as NUS. A few varieties of NUS often exhibit stable performance across many agronomic traits (Hunter et al. 2019). Considering limited genetic and economic resources, breeding can be performed in multiple biparental populations to improve specific characteristics within each population, followed by interpopulation crosses to improve multiple traits simultaneously. Furthermore, because each biparental population, developed independently, is expected to maintain distinct genetic variations, it is necessary to incorporate the effects of different alleles from each population into the UC calculations.

In this study, we assumed that different populations exhibited distinct allele effects due to differences in linkage patterns or genetic origins. To address this issue, we extended the UC calculations to incorporate population-specific marker effects. Our results reaffirm that selecting cross pairs based on UC is more effective than selecting individuals based solely on genotypic values. However, successful application in practical breeding will require further research considering additional factors, such as trait genetic architecture, appropriate selection intensity, and optimal frequency for updating genomic prediction models.

## Supplementary Information

Below is the link to the electronic supplementary material.

Supplementary file1 (DOCX 1480 KB)

Supplementary file2 (DOCX 77 KB)

Supplementary file3 (DOCX 269 KB)

Supplementary file4 (DOCX 323 KB)

## Data Availability

The datasets generated and analyzed during the current study are available in the ‘Sei-Kinoshita/RPSP’ repository on GitHub (https://github.com/Sei-Kinoshita/RPSP). Genome assembly data and predicted gene sequences were obtained from Plant GARDEN (https://plantgarden.jp/ja/list/t48386/genome/t48386.G005). The obtained genome sequence reads are available from the DDBJ Sequence Read Archive (DRA) under the accession number DRA019597. The BioProject accession number of the submitted dataset is PRJDB19161.

## References

[CR1] Allier A, Moreau L, Charcosset A, Teyssèdre S, Lehermeier C (2019) Usefulness criterion and post-selection parental contributions in multi-parental crosses: application to polygenic trait introgression. G3: Genes Genomes Genet 9(5):1469–1479. 10.1534/g3.119.400129 10.1534/g3.119.400129 PMC6505154 30819823

[CR2] Alonge M, Soyk S, Ramakrishnan S, Wang X, Goodwin S, Sedlazeck FJ, Lippman ZB, Schatz MC (2019) RaGOO: fast and accurate reference-guided scaffolding of draft genomes. Genome Biol 20:224. 10.1186/s13059-019-1829-6 31661016 10.1186/s13059-019-1829-6 PMC6816165

[CR3] Arrones A, Vilanova S, Plazas M, Mangino G, Pascual L, Díez MJ, Prohens J, Gramazio P (2020) The dawn of the age of multi-parent MAGIC populations in plant breeding: novel powerful next-generation resources for genetic analysis and selection of recombinant elite material. Biology 9(8):229. 10.3390/biology9080229 32824319 10.3390/biology9080229 PMC7465826

[CR4] Bassi FM, Bentley AR, Charmet G, Ortiz R, Crossa J (2016) Breeding schemes for the implementation of genomic selection in wheat (Triticum spp.). Plant Sci 242:23–36. 10.1016/j.plantsci.2015.08.021 26566822 10.1016/j.plantsci.2015.08.021

[CR5] Bernardo R (2008) Molecular markers and selection for complex traits in plants: Learning from the last 20 years. Crop Sci 48(5):1649–1664. 10.2135/cropsci2008.03.0131

[CR6] Bernardo R (2014) Genomewide selection of parental inbreds: Classes of loci and virtual biparental populations. Crop Sci 54(6):2586–2595. 10.2135/cropsci2014.01.0088

[CR7] Bernardo R, Yu J (2007) Prospects for genomewide selection for quantitative traits in maize. Crop Sci 47(3):1082–1090. 10.2135/cropsci2006.11.0690

[CR8] Cabanettes F, Klopp C (2018) D-GENIES: Dot plot large genomes in an interactive, efficient and simple way. PeerJ 6:e4958. 10.7717/peerj.4958 29888139 10.7717/peerj.4958 PMC5991294

[CR9] Cheng H, Concepcion GT, Feng X, Zhang H, Li H (2021) Haplotype-resolved de novo assembly using phased assembly graphs with hifiasm. Nat Methods 18(2):170–175. 10.1038/s41592-020-01056-5 33526886 10.1038/s41592-020-01056-5 PMC7961889

[CR10] Daetwyler HD, Hayden MJ, Spangenberg GC, Hayes BJ (2015) Selection on optimal haploid value increases genetic gain and preserves more genetic diversity relative to genomic selection. Genetics 200(4):1341–1348. 10.1534/genetics.115.178038 26092719 10.1534/genetics.115.178038 PMC4574260

[CR11] Danguy des Déserts AD, Durand N, Servin B, Goudemand-Dugué E, Alliot JM, Ruiz D, Charmet G, Elsen JM, Bouchet S (2023) Comparison of genomic-enabled cross selection criteria for the improvement of inbred line breeding populations. G3 (Bethesda) 13(11): 195. 10.1093/g3journal/jkad195 10.1093/g3journal/jkad195 PMC10627264 37625792

[CR12] De la Rosa PM, Mark B (2023) A telomere identification toolkit. Zenodo. 10.5281/zenodo.10091385

[CR13] Gaynor RC, Gorjanc G, Bentley AR, Ober ES, Howell P, Jackson R, Mackay IJ, Hickey JM (2017) A two-part strategy for using genomic selection to develop inbred lines. Crop Sci 57(5):2372–2386. 10.2135/cropsci2016.09.0742

[CR14] Hamazaki K, Iwata H (2020) RAINBOW: Haplotype-based genome-wide association study using a novel SNP-set method. PLoS Comput Biol 16(2):e1007663. 10.1371/journal.pcbi.1007663 32059004 10.1371/journal.pcbi.1007663 PMC7046296

[CR15] Hayes BJ, Bowman PJ, Chamberlain AJ, Goddard ME (2009) Invited review: genomic selection in dairy cattle: progress and challenges. J Dairy Sci 92(2):433–443. 10.3168/jds.2008-1646 19164653 10.3168/jds.2008-1646

[CR16] Hunter D, Borelli T, Beltrame DMO, Oliveira CNS, Coradin L, Wasike VW, Wasilwa L, Mwai J, Manjella A, Samarasinghe GWL, Madhujith T, Nadeeshani HVH, Tan A, Ay ST, Güzelsoy N, Lauridsen N, Gee E, Tartanac F (2019) The potential of neglected and underutilized species for improving diets and nutrition. Planta 250(3):709–729. 10.1007/s00425-019-03169-4 31025196 10.1007/s00425-019-03169-4

[CR17] Iwata H, Hayashi T, Terakami S, Takada N, Saito T, Yamamoto T (2013) Genomic prediction of trait segregation in a progeny population: a case study of Japanese pear (Pyrus pyrifolia). BMC Genet 14(81):81. 10.1186/1471-2156-14-81 24028660 10.1186/1471-2156-14-81 PMC3847345

[CR18] Jannink J-L, Lorenz AJ, Iwata H (2010) Genomic selection in plant breeding: from theory to practice. Brief Funct Genomics 9(2):166–177. 10.1093/bfgp/elq001 20156985 10.1093/bfgp/elq001

[CR19] Kinoshita S, Sakurai K, Hamazaki K, Tsusaka T, Sakurai M, Kurosawa T, Aoki Y, Shirasawa K, Isobe S, Iwata H (2023) Assessing the potential for genome-assisted breeding in red perilla using quantitative trait locus analysis and genomic prediction. Genes 14(12):2137. 10.3390/genes14122137 38136959 10.3390/genes14122137 PMC10742415

[CR20] Koebner R (2003) MAS in cereals: Green for maize, amber for rice, still red for wheat and barley. Marker assisted selection: a fast track to increase genetic gain in plant and animal breeding. 17–18.

[CR21] Langmead B, Salzberg SL (2012) Fast gapped-read alignment with Bowtie 2. Nat Methods 9(4):357–359. 10.1038/nmeth.1923 22388286 10.1038/nmeth.1923 PMC3322381

[CR22] Lehermeier C, Teyssèdre S, Schön C-C (2017) Genetic gain increases by applying the usefulness criterion with improved variance prediction in selection of crosses. Genetics 207(4):1651–1661. 10.1534/genetics.117.300403 29038144 10.1534/genetics.117.300403 PMC5714471

[CR23] Li W, Boer MP, Zheng C, Joosen RVL, Van Eeuwijk FA (2021) An IBD-based mixed model approach for QTL mapping in multiparental populations. Theor Appl Genet 134(11):3643–3660. 10.1007/s00122-021-03919-7 34342658 10.1007/s00122-021-03919-7 PMC8519866

[CR24] Mahadevaiah C, Appunu C, Aitken K, Suresha GS, Vignesh P, Mahadeva Swamy HK, Valarmathi R, Hemaprabha G, Alagarasan G, Ram B (2021) Genomic selection in sugarcane: current status and future prospects. Front Plant Sci 12:708233. 10.3389/fpls.2021.708233 34646284 10.3389/fpls.2021.708233 PMC8502939

[CR25] Mangino G, Arrones A, Plazas M, Pook T, Prohens J, Gramazio P, Vilanova S (2022) Newly developed MAGIC population allows identification of strong associations and candidate genes for anthocyanin pigmentation in eggplant. Front Plant Sci 13:847789. 10.3389/fpls.2022.847789 35330873 10.3389/fpls.2022.847789 PMC8940277

[CR26] Maurer A, Sannemann W, Léon J, Pillen K (2017) Estimating parent-specific QTL effects through cumulating linked identity-by-state SNP effects in multiparental populations. Heredity 118(5):477–485. 10.1038/hdy.2016.121 27966535 10.1038/hdy.2016.121 PMC5520528

[CR27] Meuwissen THE, Hayes BJ, Goddard ME (2001) Prediction of total genetic value using genome-wide dense marker maps. Genetics 157(4):1819–1829. 10.1093/genetics/157.4.1819 11290733 10.1093/genetics/157.4.1819 PMC1461589

[CR28] Mohammadi M, Tiede T, Smith KP (2015) PopVar: a genome-wide procedure for predicting genetic variance and correlated response in biparental breeding populations. Crop Sci 55(5):2068–2077. 10.2135/cropsci2015.01.0030

[CR29] Müller D, Schopp P, Melchinger AE (2018) Selection on expected maximum haploid breeding values can increase genetic gain in recurrent genomic selection|Genomes|Genetics. G3 (Bethesda) 8(4):1173–1181. 10.1534/g3.118.200091 29434032 10.1534/g3.118.200091 PMC5873908

[CR30] Pérez P, De Los CG (2014) Genome-wide regression and prediction with the BGLR statistical package. Genetics 198(2):483–495. 10.1534/genetics.114.164442 25009151 10.1534/genetics.114.164442 PMC4196607

[CR31] Ramalingam J, Raveendra C, Savitha P, Vidya V, Chaithra TL, Velprabakaran S, Saraswathi R, Ramanathan A, Arumugam Pillai MP, Arumugachamy S, Vanniarajan C (2020) Gene pyramiding for achieving enhanced resistance to bacterial blight, blast, and sheath blight diseases in rice. Front Plant Sci 11:591457. 10.3389/fpls.2020.591457 33329656 10.3389/fpls.2020.591457 PMC7711134

[CR32] Ranallo-Benavidez TR, Jaron KS, Schatz MC (2020) GenomeScope 2.0 and Smudgeplot for reference-free profiling of polyploid genomes. Nat Commun 11(1):1432. 10.1038/s41467-020-14998-3 32188846 10.1038/s41467-020-14998-3 PMC7080791

[CR33] Rembe M, Zhao Y, Wendler N, Oldach K, Korzun V, Reif JC (2022) The potential of genome-wide prediction to support parental selection, evaluated with data from a commercial barley breeding program. Plants 11(19):2564. 10.3390/plants11192564 36235430 10.3390/plants11192564 PMC9571379

[CR34] Sakurai K, Hamazaki K, Inamori M, Kaga A, Iwata H (2024) Cross potential selection: a proposal for optimizing crossing combinations in recurrent selection using the usefulness criterion of future inbred lines. G3 Genes Genomes Genetics. 10.1093/g3journal/jkae224 39312266 10.1093/g3journal/jkae224 PMC11540310

[CR35] Sanchez D, Sadoun SB, Mary-Huard T, Allier A, Moreau L, Charcosset A (2023) Improving the use of plant genetic resources to sustain breeding programs’ efficiency. Proc Natl Acad Sci USA 120(14):e2205780119. 10.1073/pnas.2205780119 36972431 10.1073/pnas.2205780119 PMC10083577

[CR36] Shirasawa K, Hirakawa H, Isobe S (2016) Analytical workflow of double-digest restriction site-associated DNA sequencing based on empirical and in silico optimization in tomato. DNA Res 23(2):145–153. 10.1093/dnares/dsw004 26932983 10.1093/dnares/dsw004 PMC4833422

[CR37] Simão FA, Waterhouse RM, Ioannidis P, Kriventseva EV, Zdobnov EM (2015) BUSCO: assessing genome assembly and annotation completeness with single-copy orthologs. Bioinformatics 31(19):3210–3212. 10.1093/bioinformatics/btv351 26059717 10.1093/bioinformatics/btv351

[CR38] Stiehler F, Steinborn M, Scholz S, Dey D, Weber APM, Denton AK (2021) Helixer: cross-species gene annotation of large eukaryotic genomes using deep learning. Bioinformatics 36(22–23):5291–5298. 10.1093/bioinformatics/btaa1044 33325516 10.1093/bioinformatics/btaa1044 PMC8016489

[CR39] Tamura K, Sakamoto M, Tanizawa Y, Mochizuki T, Matsushita S, Kato Y, Ishikawa T, Okuhara K, Nakamura Y, Bono H (2023) A highly contiguous genome assembly of red perilla (*Perilla frutescens*) domesticated in Japan. DNA Res 30(1):dsac044. 10.1093/dnares/dsac044 36383440 10.1093/dnares/dsac044 PMC9835750

[CR40] Wang F, Feldmann MJ, Runcie DE (2024) Why usefulness is rarely useful, G3: Genes. Genomes, Genetics 15(3):jkae296. 10.1093/g3journal/jkae296 10.1093/g3journal/jkae296 PMC11917469 39719001

[CR42] Yamamoto E, Matsunaga H, Onogi A, Kajiya-Kanegae H, Minamikawa M, Suzuki A, Shirasawa K, Hirakawa H, Nunome T, Yamaguchi H, Miyatake K, Ohyama A, Iwata H, Fukuoka H (2016) A simulation-based breeding design that uses whole-genome prediction in tomato. Sci Rep 6(1):19454. 10.1038/srep19454 26787426 10.1038/srep19454 PMC4726135

[CR43] Ye CY, Fan L (2021) Orphan crops and their wild relatives in the genomic era. Mol Plant 14(1):27–39. 10.1016/j.molp.2020.12.013 33346062 10.1016/j.molp.2020.12.013

[CR44] Zhong S, Jannink JL (2007) Using quantitative trait loci results to discriminate among crosses on the basis of their progeny mean and variance. Genetics 177(1):567–576. 10.1534/genetics.107.075358 17660556 10.1534/genetics.107.075358 PMC2013701

