# Supplementary material for: Optimization of crossing strategy based on the usefulness criterion in interpopulation crosses considering different marker effects among populations
Source: Theor Appl Genet. 2025 Jun 20;138(7):155. doi: 10.1007/s00122-025-04935-7 (PMC12178988; doi:10.1007/s00122-025-04935-7)
Supplement: Supplementary file 1 — Supplementary file1 (DOCX 1480 KB) [file 122_2025_4935_MOESM1_ESM.docx]

Supplementary information for

**Optimization of Crossing Strategy Using the Usefulness Criterion in Inter-population Crosses Considering Different Genetic Effects Among Populations**

**Sei Kinoshita ^1^, Kengo Sakurai ^1^, Kosuke Hamazaki ^2^, Takahiro Tsusaka ^3^, Miki Sakurai ^3^, Kenta Shirasawa ^4^, Sachiko Isobe ^1^, and Hiroyoshi Iwata ^1,^***

^1^ Graduate School of Agricultural and Life Sciences, University of Tokyo, Tokyo, Japan

^2^ RIKEN Center for Advanced Intelligence Project, Chiba, Japan

^3^ TSUMURA & CO., Ibaraki, Japan

^3^ Kazusa DNA Research Institute, Chiba, Japan

*** Correspondence:**Corresponding Author
[hiroiwata@g.ecc.u-tokyo.ac.jp](mailto:hiroiwata@g.ecc.u-tokyo.ac.jp)


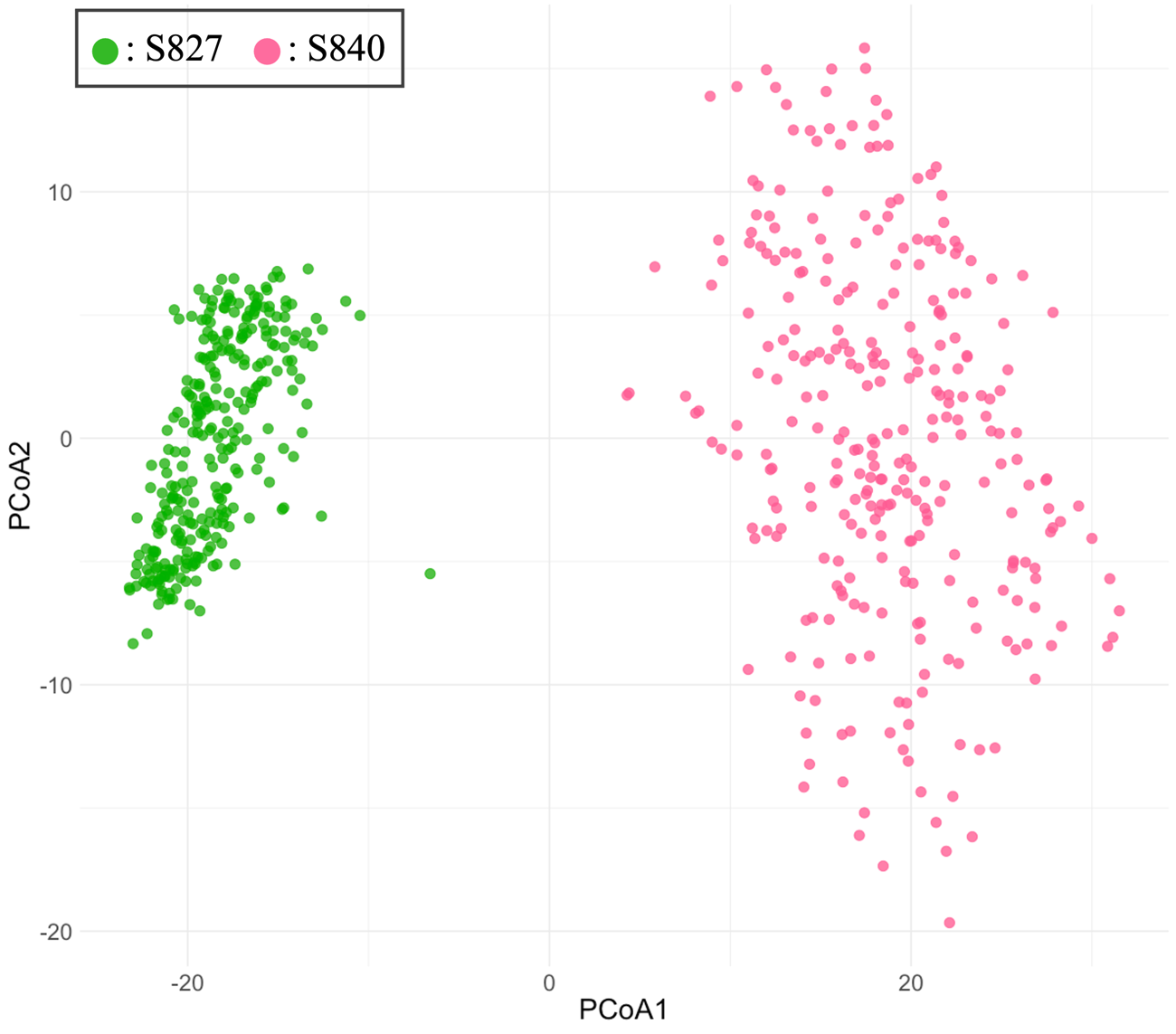
**Fig. S1** PCoA plot based on Euclidean distance matrix of genome data. Green points: individuals in S827; pink points: individuals in S840.

**Fig. S2** Estimated marker effects of each population and trait. The left and right panels show the marker effects in two biparental populations, i.e., S827 and S840, respectively. The marker effects of all 1,951 single nucleotide polymorphisms are shown; however, the effects of markers that are not polymorphic within each population are set to zero
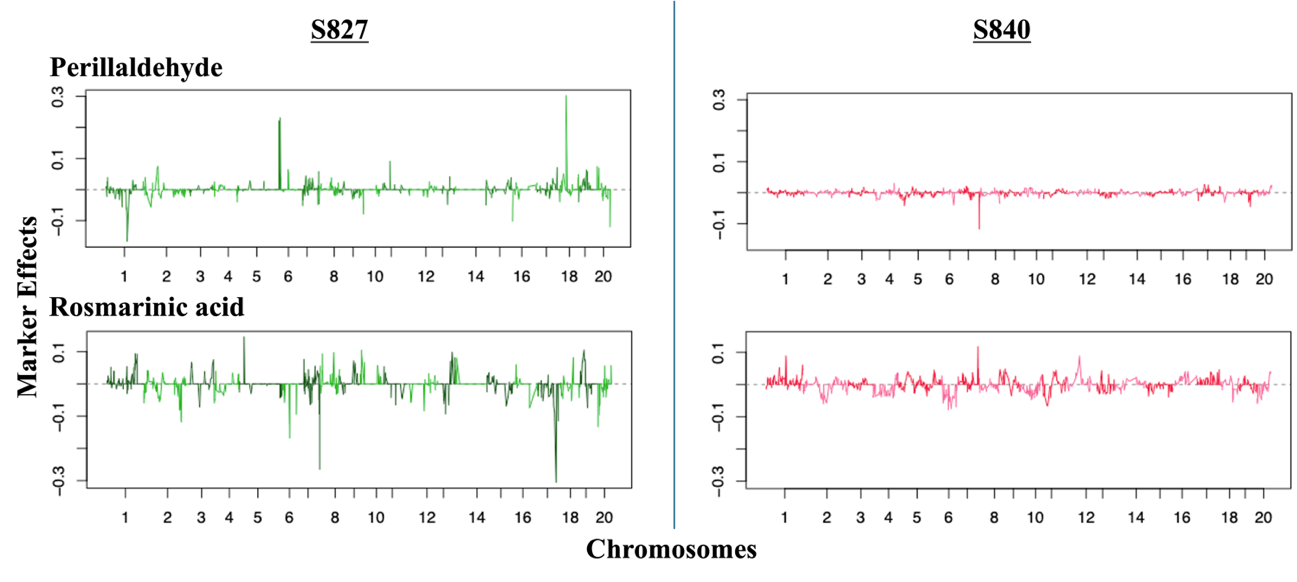
.


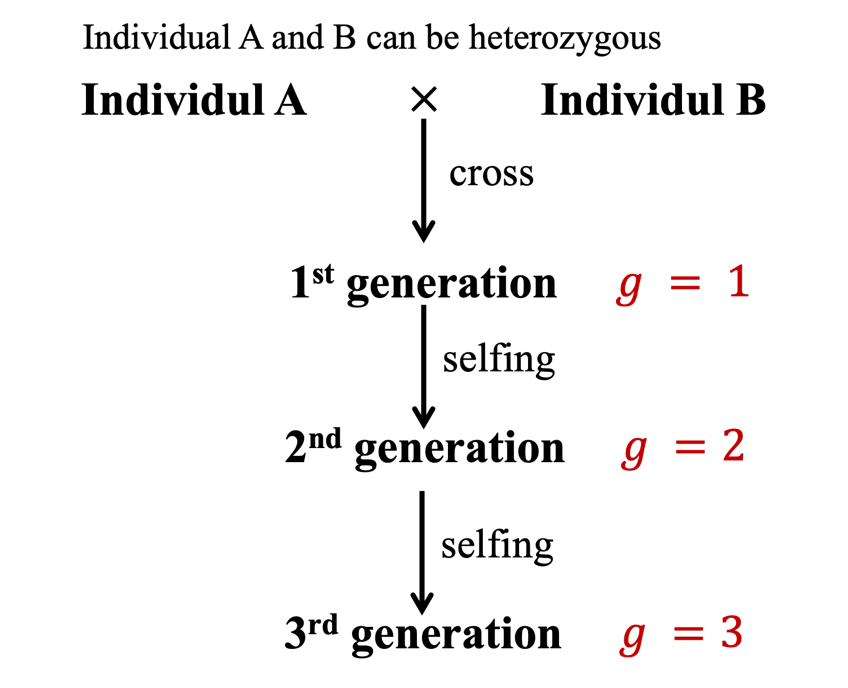


**Fig. S3** Illustration of the generation represented by $g$ in Equation 9.


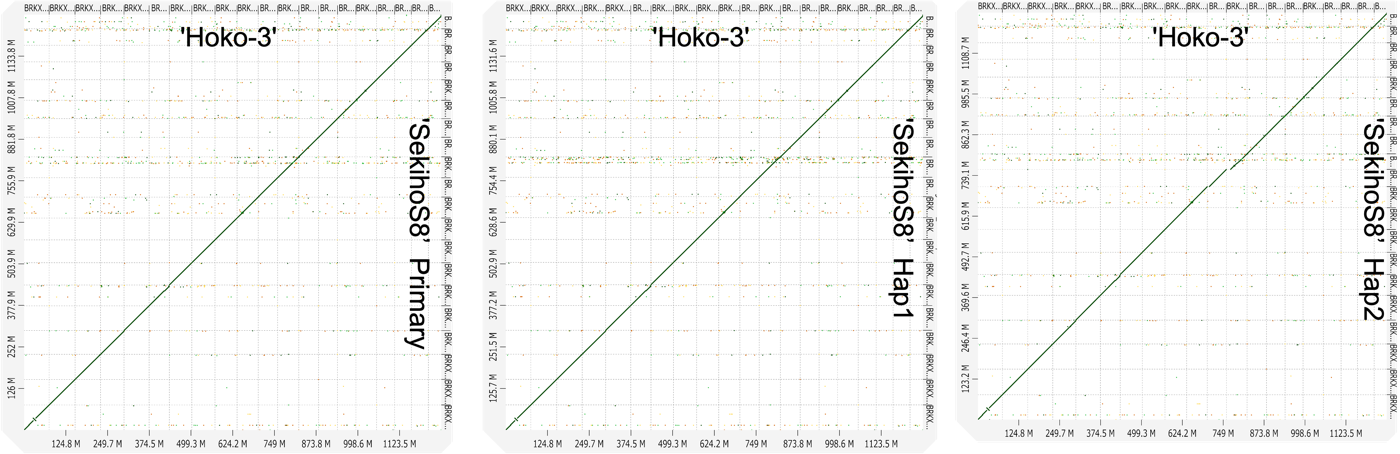


**Fig. S4** Comparison of the genome structures of 'Hoko-3' and 'SekihoS8'.


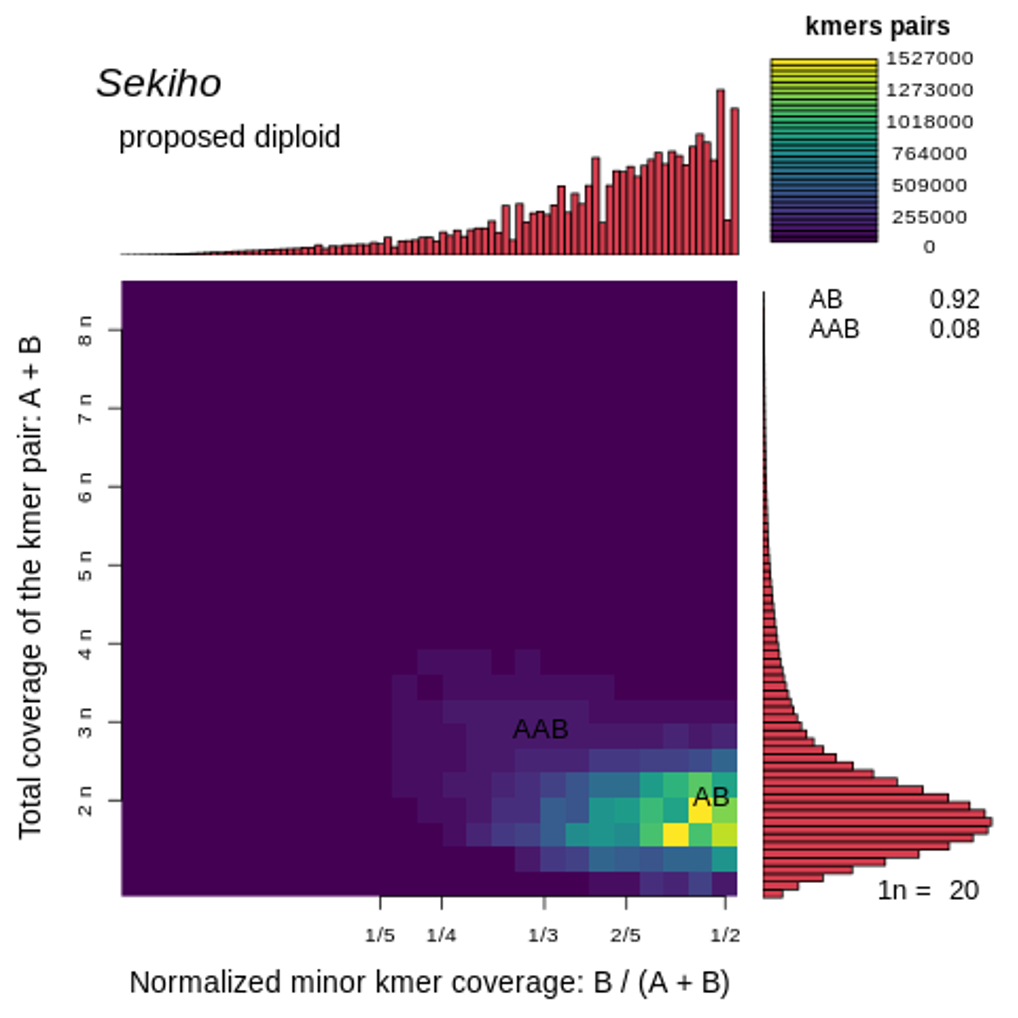
**Fig. S5** Genome structure estimation of 'SekihoS8' using Smudgeplot. The analysis was conducted using the primary assembly.


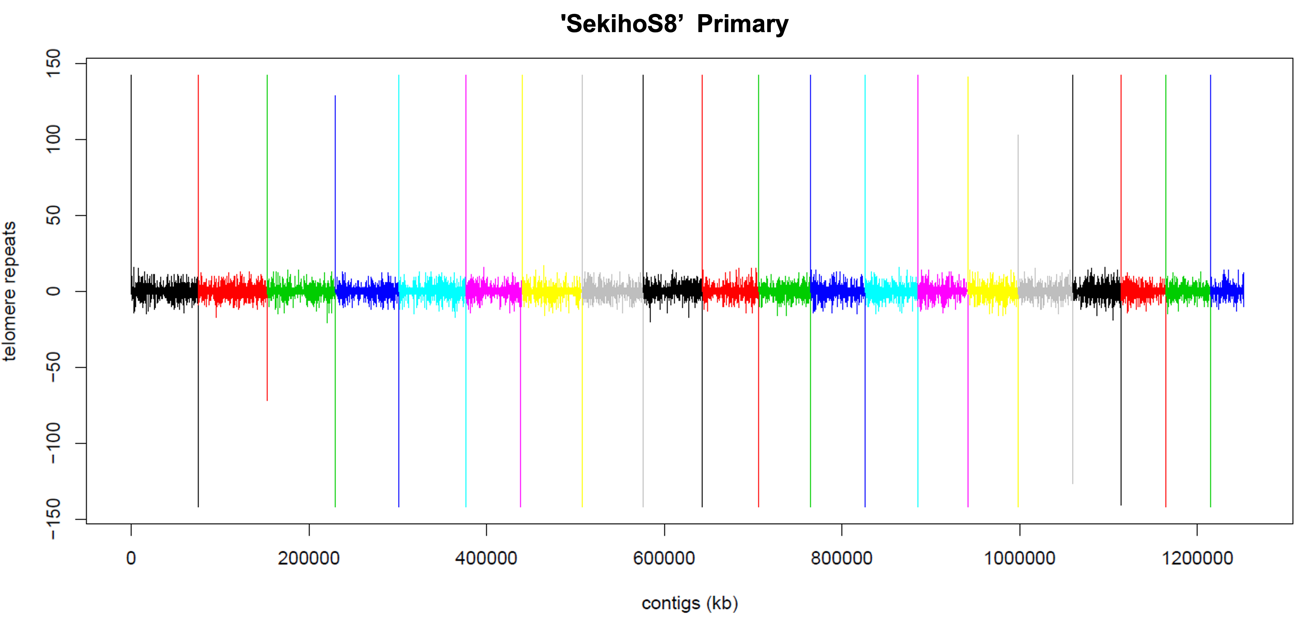
**Fig. S6a** Results of telomere sequence detection on the 'SekihoS8’ primary genome

**Fig. S6b** Results of telomere sequence detection on the 'SekihoS8’ Hap1 genome
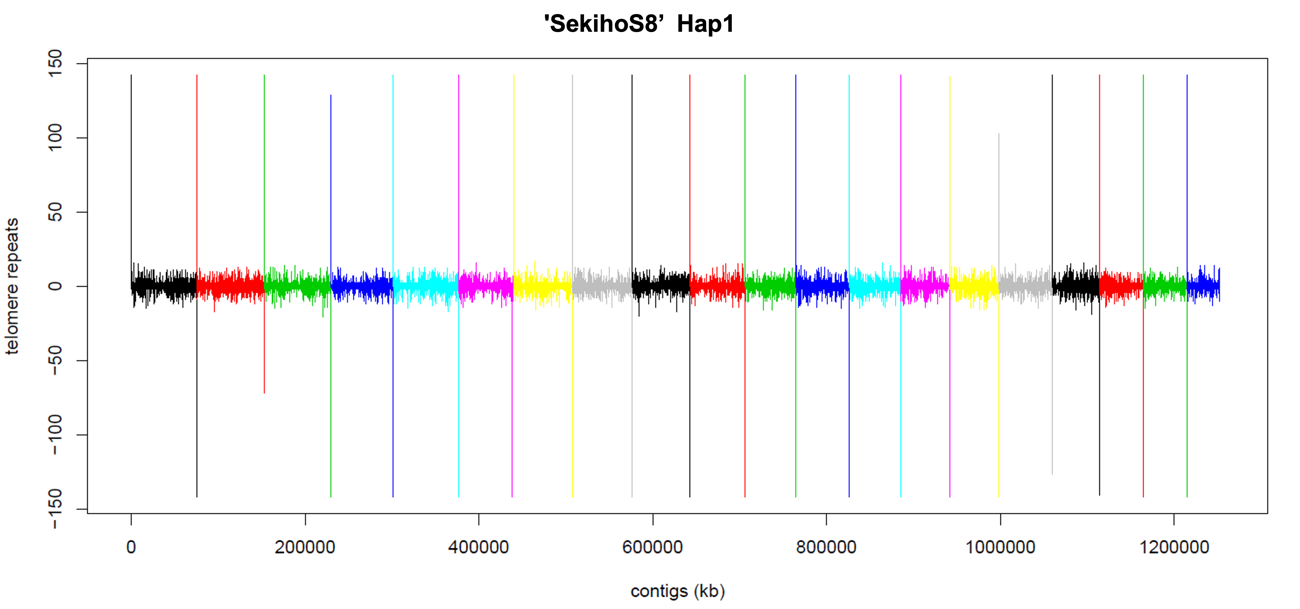


**Fig. S6c** Results of telomere sequence detection on the 'SekihoS8’ Hap2 genome
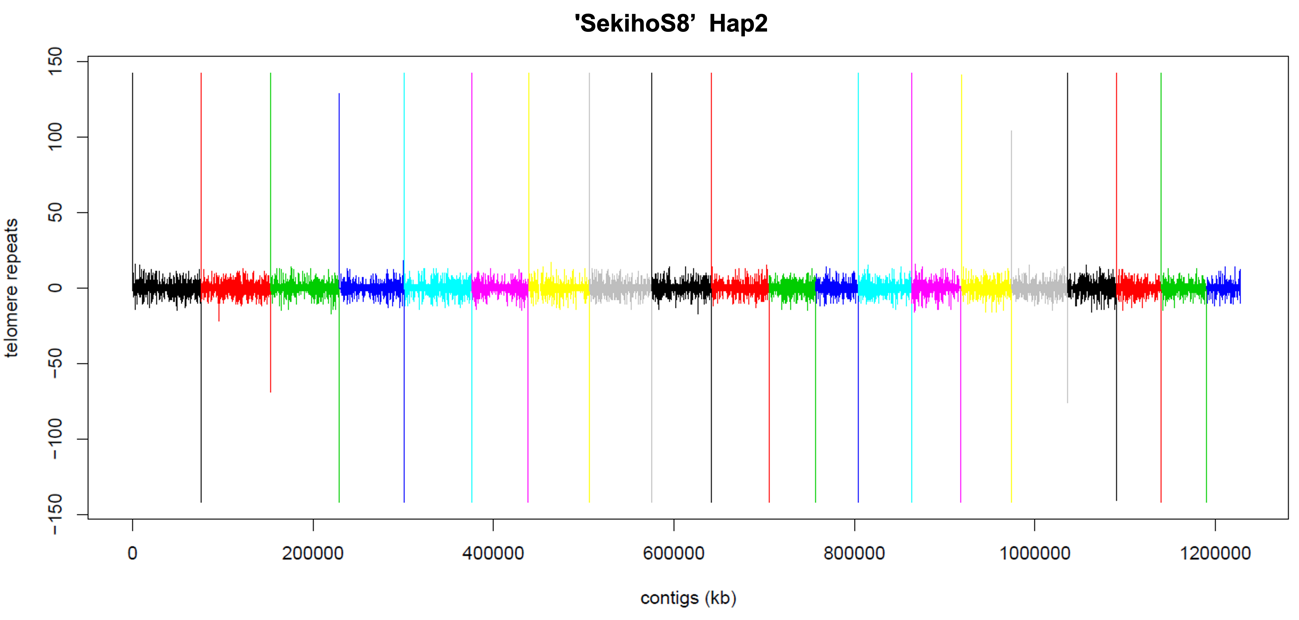


**
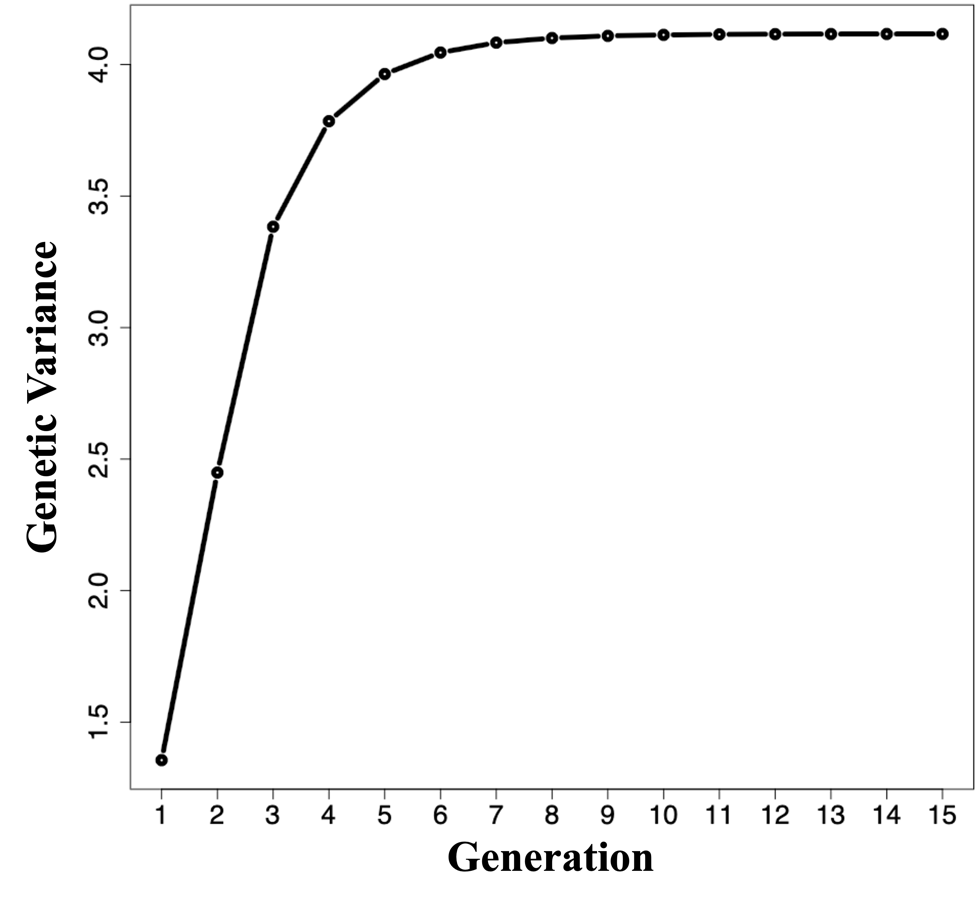
**

**Fig. S7** Change in genetic variance during inbreeding via single-seed descent (SSD) repeated after a single cross.

**Supplementary Table S1. Statistics of the assembled 'SekihoS8' genome.**

| Result of assembly by hifiasm | | | |
| --- | --- | --- | --- |
|  | Hap1 | Hap2 | Primary |
| Number of sequences | 357 | 161 | 343 |
| Total length (bp) | 1,268,085,799 | 1,235,131,714 | 1,270,581,718 |
| Average length (bp) | 3,552,061 | 7,671,626 | 3,704,320 |
| Max length (bp) | 77,417,536 | 77,371,512 | 77,417,536 |
| Min length (bp) | 20,070 | 21,559 | 20,070 |
| N50 length (bp) | 55,681,988 | 55,476,749 | 55,956,609 |
| After removal of candidate chloroplast and mitochondrial genome sequences | | | |
|  | Hap1 | Hap2 | Primary |
| Number of sequences | 121 | 106 | 108 |
| Total length (bp) | 1,257,287,594 | 1,231,835,063 | 1,259,745,714 |
| Average length (bp) | 10,390,807 | 11,621,086 | 11,664,312 |
| Max length (bp) | 77,417,536 | 77,371,512 | 77,417,536 |
| Min length (bp) | 21,559 | 21,559 | 21,559 |
| N50 length (bp) | 55,681,988 | 55,476,749 | 55,956,609 |
| Aligned to Pfru_yukari_1.0 using Ragoo | | | |
|  | Pfru_SekihoH1_1.0 | Pfru_SekihoH2_1.0 | Pfru_SekihoUp_1.0 |
| Number of sequences | 20 | 20 | 20 |
| Total length (bp) | 1,257,297,694 | 1,231,843,663 | 1,259,754,514 |
| Average length (bp) | 62,864,885 | 61,592,183 | 62,987,726 |
| Max length (bp) | 77,417,536 | 77,371,512 | 77,417,536 |
| Min length (bp) | 42,555,208 | 41,231,751 | 42,872,164 |
| N50 length (bp) | 63,696,810 | 63,607,629 | 63,696,810 |
| BUSCO v5.2.2 (odb10, %, n = 1,614) | | | |
|  | Pfru_SekihoH1_1.0 | Pfru_SekihoH2_1.0 | Pfru_SekihoUp_1.0 |
| Complete | 99.4 | 99.4 | 99.4 |
| Single | 4.2 | 9.7 | 4.2 |
| Duplicate | 95.2 | 89.7 | 95.2 |
| Fragment | 0.2 | 0.2 | 0.2 |
| Missing | 0.4 | 0.4 | 0.4 |

**Supplementary Table S2. Statistics of the predicted genes on the 'SekihoS8' genomes.**

| Result of assembly by hifiasm | | | |
| --- | --- | --- | --- |
|  | Pfru_SekihoH1 | Pfru_SekihoH2 | Pfru_SekihoUp |
| Number of sequences | 65,078 | 62,895 | 65,161 |
| Total length (bp) | 81,205,836 | 78,622,701 | 81,312,075 |
| Average length (bp) | 1,248 | 1,250 | 1,248 |
| Max length (bp) | 16,365 | 16,368 | 16,365 |
| Min length (bp) | 102 | 102 | 102 |
| N50 length (bp) | 1,632 | 1,635 | 1,632 |
| BUSCO v5.2.2 (odb10, %, n = 1,614) | | | |
|  | Pfru_SekihoH1_1.0.fasta | Pfru_SekihoH2_1.0.fasta | Pfru_SekihoUp_1.0.fasta |
| Complete | 99.1 | 98.7 | 99.1 |
| Single | 4.2 | 9.7 | 4.2 |
| Duplicate | 94.9 | 89.0 | 94.9 |
| Fragment | 0.6 | 0.7 | 0.6 |
| Missing | 0.3 | 0.6 | 0.3 |

**Supplementary Table S3. The number of replicates where Scenario 3 exceeded Scenario 2 in G_15_ for mean genetic gains of the entire population, top 1% of individuals, and genetic variance.**

| $t=1$ | | | |
| --- | --- | --- | --- |
|  | Perillaldehyde | Rosmarinic Acid | Selection Index |
| Mean of the entire population | 0 | 50*** | 3 |
| Mean of the top 1% individuals | 1 | 50*** | 50*** |
| Genetic variance | 49*** | 50*** | 50*** |
| $t=2$ | | | |
|  | Perillaldehyde | Rosmarinic Acid | Selection Index |
| Mean of the entire population | 4 | 47*** | 34*** |
| Mean of the top 1% individuals | 11 | 48*** | 43*** |
| Genetic variance | 38*** | 40*** | 49*** |
| $t=3$ | | | |
|  | Perillaldehyde | Rosmarinic Acid | Selection Index |
| Mean of the entire population | 9 | 43*** | 41*** |
| Mean of the top 1% individuals | 17 | 44*** | 48*** |
| Genetic variance | 42*** | 37*** | 47*** |
| $t=4$ | | | |
|  | Perillaldehyde | Rosmarinic Acid | Selection Index |
| Mean of the entire population | 6 | 46*** | 35*** |
| Mean of the top 1% individuals | 11 | 46*** | 45*** |
| Genetic variance | 40*** | 38*** | 48*** |

Here, $t$ indicates the generation of second-round cross. Cells marked with "***" indicate cases where Scenario 3 outperformed Scenario 2 a significantly greater number of times, based on a binomial test (significance level: p < 0.01).
