# Supplementary material for: Optimization of crossing strategy based on the usefulness criterion in interpopulation crosses considering different marker effects among populations
Source: Theor Appl Genet. 2025 Jun 20;138(7):155. doi: 10.1007/s00122-025-04935-7 (PMC12178988; doi:10.1007/s00122-025-04935-7)
Supplement: Supplementary file 2 — Supplementary file2 (DOCX 77 KB) [file 122_2025_4935_MOESM2_ESM.docx]

Supplementary information for

**Optimization of Crossing Strategy Using the Usefulness Criterion in Inter-population Crosses Considering Different Genetic Effects Among Populations**

**Sei Kinoshita ^1^, Kengo Sakurai ^1^, Kosuke Hamazaki ^2^, Takahiro Tsusaka ^3^, Miki Sakurai ^3^, Terue Kurosawa ^3^, Youichi Aoki ^3^, Kenta Shirasawa ^4^, Sachiko Isobe ^1^, and Hiroyoshi Iwata ^1,^***

^1^ Graduate School of Agricultural and Life Sciences, University of Tokyo, Tokyo, Japan

^2^ RIKEN Center for Advanced Intelligence Project, Chiba, Japan

^3^ TSUMURA & CO., Ibaraki, Japan

^3^ Kazusa DNA Research Institute, Chiba, Japan

*** Correspondence:**Corresponding Author
[hiroiwata@g.ecc.u-tokyo.ac.jp](mailto:hiroiwata@g.ecc.u-tokyo.ac.jp)

**Supplementary File 2**

This supplementary file describes the methods and results of a comparison between genomic prediction (GP) models considering only additive effects and those incorporating both additive and dominant effects, using the S827 and S840 populations separately.

The construction of the GP model considering only additive effects (hereafter referred to as the A model) follows the description provided in the "GP model" section of the main text. For the model incorporating dominant effects (hereafter referred to as the AD model), we used the same BayesB approach as in the A model. The model is represented as follows:

$\boldsymbol{y}_{\boldsymbol{pm}}\boldsymbol{=}\boldsymbol{\mu}_{\boldsymbol{pm}}\boldsymbol{+}\mathbf{X}_{\boldsymbol{p}}\boldsymbol{\beta}_{\boldsymbol{pm}}\boldsymbol{+}\mathbf{Z}_{\boldsymbol{p}}\boldsymbol{\delta}_{\boldsymbol{pm}}\boldsymbol{+}\boldsymbol{\varepsilon}_{\boldsymbol{pm}}$, (2)

where $N$ is the number of individuals in one population, $L$ is the number of markers, $\boldsymbol{y}_{\boldsymbol{pm}}$ is an $N\times1$ vector representing phenotypic values for the $m$^th^ trait in the $p$^th^ population, $\mu_{pm}$ is the overall mean, $\mathbf{X}_{\boldsymbol{p}}$ and $\mathbf{Z}_{\boldsymbol{p}}$ are $N\times L$ matrices representing the additive and dominant genotype indicators for the $p$^th^ population, respectively, $\boldsymbol{\beta}_{pm}$ and $\boldsymbol{\delta}_{\boldsymbol{pm}}$ are $L\times1$ vectors corresponding to marker effects for the $m$^th^ trait and $p$^th^ population, respectively, and $\boldsymbol{\varepsilon}_{pm}\sim N(0, I\sigma_{e}^{2})$ is an $N\times1$ vector of errors, where $\sigma_{e}^{2}$ is the error variance. The model was implemented using the “BGLR” function in the “BGLR” package version 1.1.0 in R (Pérez and De Los Campos 2014). As with the A model, the Markov Chain Monte Carlo was run for 60,000 iterations, with the first 12,000 samples discarded as burn-in and a sampling interval (thinning) of 5.

To evaluate the GP accuracy, a 10-fold cross validation was performed and repeated 10 times for both A model and AD model. For each cross validation, the Pearson correlation between the observed and predicted values was calculated, and the average of these correlations was used as the prediction accuracy. The prediction accuracy of A model and AD model is shown in the figure below.


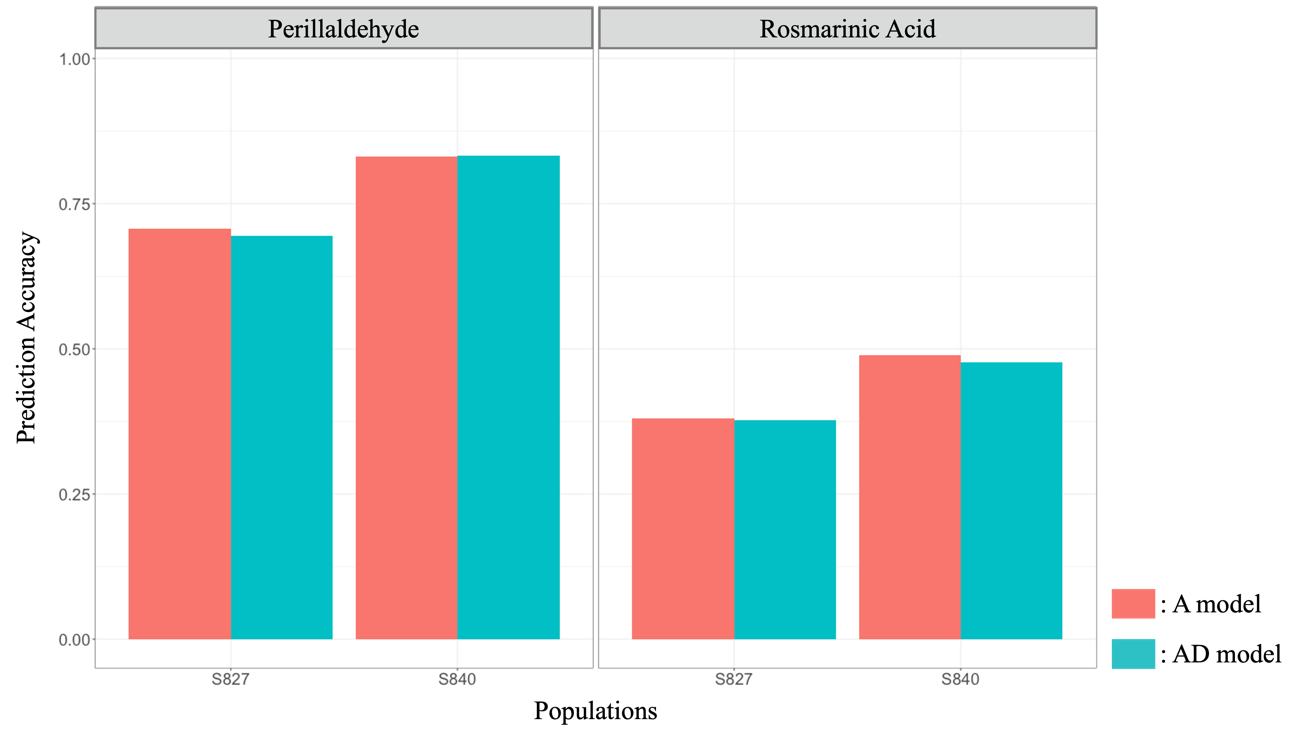
For perillaldehyde, the prediction accuracy of the AD model (0.833) slightly exceeded that of the A model (0.832) only in the S840 population. In other cases, A model slightly exceeded AD model.
