# Supplementary material for: Optimization of crossing strategy based on the usefulness criterion in interpopulation crosses considering different marker effects among populations
Source: Theor Appl Genet. 2025 Jun 20;138(7):155. doi: 10.1007/s00122-025-04935-7 (PMC12178988; doi:10.1007/s00122-025-04935-7)
Supplement: Supplementary file 3 — Supplementary file3 (DOCX 269 KB) [file 122_2025_4935_MOESM3_ESM.docx]

Supplementary information for

**Optimization of Crossing Strategy Using the Usefulness Criterion in Inter-population Crosses Considering Different Genetic Effects Among Populations**

**Sei Kinoshita ^1^, Kengo Sakurai ^1^, Kosuke Hamazaki ^2^, Takahiro Tsusaka ^3^, Miki Sakurai ^3^, Kenta Shirasawa ^4^, Sachiko Isobe ^1^, and Hiroyoshi Iwata ^1,^***

^1^ Graduate School of Agricultural and Life Sciences, University of Tokyo, Tokyo, Japan

^2^ RIKEN Center for Advanced Intelligence Project, Chiba, Japan

^3^ TSUMURA & CO., Ibaraki, Japan

^3^ Kazusa DNA Research Institute, Chiba, Japan

*** Correspondence:**Corresponding Author
[hiroiwata@g.ecc.u-tokyo.ac.jp](mailto:hiroiwata@g.ecc.u-tokyo.ac.jp)

**Supplementary File 3**

This supplementary file describes how we tracked alleles originating from different populations. The R code used for the genome simulation is available in the “Sei-Kinoshita/RPSP” repository on GitHub (https://github.com/Sei-Kinoshita/RPSP). Here, we provide a conceptual illustration of the procedures implemented in the R code to facilitate understanding of the genome simulation workflow.

To enable tracking of alleles originating from different populations, we extended the haplotype matrix in a straightforward manner by increasing its dimensions according to the number of the original parents (the number of alleles) involved in the crosses. In this study, the two bi-parental populations, S827 and S840, were derived from the crosses 'SekihoS8' × 'st27' and 'SekihoS8' × 'st40', respectively. Because both populations share 'SekihoS8' as a common parent and the genotyping reference sequence is also based on 'SekihoS8', a haplotype score of 0 represents the allele identical to the reference ('SekihoS8'). Unlike in the case of a single bi-parental population, a haplotype score of 1 in this context represents two distinct alleles—those derived from 'st27' and 'st40', respectively. To distinguish between these two, we extended the haplotype matrix to allow for the representation of both types of score 1 alleles.

The conceptual diagram below illustrates how recombination occurs based on known parental haplotypes, how gametes are generated, how next-generation individuals are formed, and how their genotypic values are subsequently calculated.


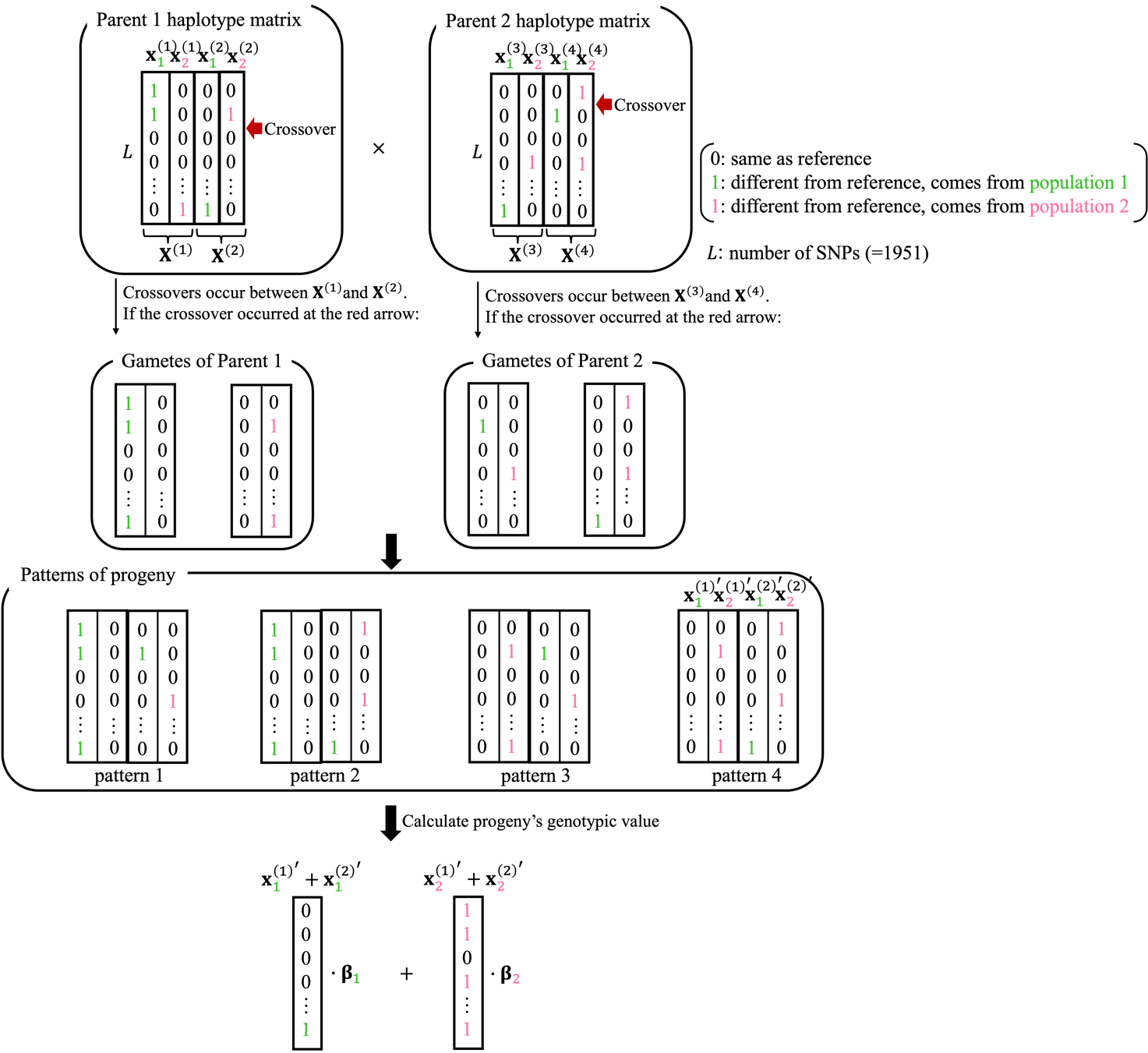


In the conceptual diagram, Parent 1 and Parent 2 represent the individuals selected as mating pairs during the first- and second-round crosses in this study. These individuals are assumed to be heterozygous. The haplotypes of Parent 1 are denoted as $\mathbf{X}^{\left( 1 \right)}$ and $\mathbf{X}^{\left( 2 \right)}$, each further divided into $\mathbf{x}_{1}^{\left( 1 \right)}$ and $\mathbf{x}_{2}^{\left( 1 \right)}$, and $\mathbf{x}_{1}^{\left( 2 \right)}$ and $\mathbf{x}_{2}^{\left( 2 \right)}$, respectively, to indicate alleles originating from different populations.

Specifically, $\mathbf{x}_{1}^{\left( 1 \right)}$ represents alleles in haplotype 1 of Parent 1 that originated from population 1 (denoted as 1), while $\mathbf{x}_{2}^{\left( 1 \right)}$ captures alleles in the same haplotype 1 that originated from population 2. Similarly, $\mathbf{x}_{1}^{\left( 2 \right)}$ and $\mathbf{x}_{2}^{\left( 2 \right)}$ correspond to population-specific alleles in haplotype 2 of Parent 1. The haplotypes of Parent 2 ($\mathbf{X}^{\left( 3 \right)}$ and $\mathbf{X}^{\left( 4 \right)}$) are represented in the same manner, as $\mathbf{x}_{1}^{\left( 3 \right)}$, $\mathbf{x}_{2}^{\left( 3 \right)}$, $\mathbf{x}_{1}^{\left( 4 \right)}$, and $\mathbf{x}_{2}^{\left( 4 \right)}$. Here, $\mathbf{X}^{\left( 1 \right)}$, $\mathbf{X}^{\left( 2 \right)}$, $\mathbf{X}^{\left( 3 \right)}$, and $\mathbf{X}^{\left( 4 \right)}$ are $L\times2$ matrices, $L=1951$ is the number of markers, $\mathbf{x}_{1}^{\left( 1 \right)}$, $\mathbf{x}_{2}^{\left( 1 \right)}$, $\mathbf{x}_{1}^{\left( 2 \right)}$, $\mathbf{x}_{2}^{\left( 2 \right)}$, $\mathbf{x}_{1}^{\left( 3 \right)}$, $\mathbf{x}_{2}^{\left( 3 \right)}$, $\mathbf{x}_{1}^{\left( 4 \right)}$, and $\mathbf{x}_{2}^{\left( 4 \right)}$ are $L\times1$ vectors.

During recombination, crossovers occur between $\mathbf{X}^{\left( 1 \right)}$ and $\mathbf{X}^{\left( 2 \right)}$, and between $\mathbf{X}^{\left( 3 \right)}$ and $\mathbf{X}^{\left( 4 \right)}$, determining the gametes transmitted from Parent 1 and Parent 2. The haplotypes of the next-generation individuals are thus formed based on the positions of recombination. For example, if one next-generation individual have haplotype 1 represented as $\mathbf{x}_{1}^{\left( 1 \right)^{\boldsymbol{'}}}$ and $\mathbf{x}_{2}^{\left( 1 \right)^{\boldsymbol{'}}}$, and haplotype 2 as $\mathbf{x}_{1}^{\left( 2 \right)^{\boldsymbol{'}}}$ and $\mathbf{x}_{2}^{\left( 2 \right)^{\boldsymbol{'}}}$, where $\mathbf{x}_{1}^{\left( 1 \right)^{\boldsymbol{'}}}$ and $\mathbf{x}_{2}^{\left( 1 \right)^{\boldsymbol{'}}}$ store alleles originating from population 1, and $\mathbf{x}_{1}^{\left( 2 \right)^{\boldsymbol{'}}}$ and $\mathbf{x}_{2}^{\left( 2 \right)^{\boldsymbol{'}}}$ store alleles from population 2. The genotypic value of this next-generation individual is then calculated as:

$$\left( \mathbf{x}_{1}^{\left( 1 \right)^{\boldsymbol{'}}}+\mathbf{x}_{1}^{\left( 2 \right)^{\boldsymbol{'}}} \right)\boldsymbol{\cdot}\boldsymbol{\beta}_{1}\boldsymbol{+}\left( \mathbf{x}_{2}^{\left( 1 \right)^{\boldsymbol{'}}}+\mathbf{x}_{2}^{\left( 2 \right)^{\boldsymbol{'}}} \right)\boldsymbol{\cdot}\boldsymbol{\beta}_{2}$$

where $\boldsymbol{\beta}_{1}$​ and $\boldsymbol{\beta}_{2}$ are $L\times1$ vectors representing the marker effects estimated from population 1 and population 2, respectively.
