# Supplementary material for: Optimization of crossing strategy based on the usefulness criterion in interpopulation crosses considering different marker effects among populations
Source: Theor Appl Genet. 2025 Jun 20;138(7):155. doi: 10.1007/s00122-025-04935-7 (PMC12178988; doi:10.1007/s00122-025-04935-7)
Supplement: Supplementary file 4 — Supplementary file4 (DOCX 323 KB) [file 122_2025_4935_MOESM4_ESM.docx]

Supplementary information for

**Optimization of Crossing Strategy Using the Usefulness Criterion in Inter-population Crosses Considering Different Genetic Effects Among Populations**

**Sei Kinoshita ^1^, Kengo Sakurai ^1^, Kosuke Hamazaki ^2^, Takahiro Tsusaka ^3^, Miki Sakurai ^3^, Kenta Shirasawa ^4^, Sachiko Isobe ^1^, and Hiroyoshi Iwata ^1,^***

^1^ Graduate School of Agricultural and Life Sciences, University of Tokyo, Tokyo, Japan

^2^ RIKEN Center for Advanced Intelligence Project, Chiba, Japan

^3^ TSUMURA & CO., Ibaraki, Japan

^3^ Kazusa DNA Research Institute, Chiba, Japan

*** Correspondence:**Corresponding Author
[hiroiwata@g.ecc.u-tokyo.ac.jp](mailto:hiroiwata@g.ecc.u-tokyo.ac.jp)

**Supplementary File 4**

In this Supplementary File, we present a worked toy example to facilitate the understanding of $\mathbf{x}_{1}^{\left( 1 \right)}$, $\mathbf{x}_{1}^{\left( 2 \right)}$, $\mathbf{x}_{2}^{\left( 1 \right)}$, $\mathbf{x}_{2}^{\left( 2 \right)}$, $\mathbf{x}_{1}^{\left( 3 \right)}$, $\mathbf{x}_{1}^{\left( 4 \right)}$, $\mathbf{x}_{2}^{\left( 3 \right)}$, $\mathbf{x}_{2}^{\left( 4 \right)}$, $\boldsymbol{\beta}_{1m}$ and $\boldsymbol{\beta}_{2m}$ appearing in Equations 6 and 7 of the main text. As stated in the main text, $\mathbf{x}_{1}^{\left( 1 \right)}$, $\mathbf{x}_{1}^{\left( 2 \right)}$, $\mathbf{x}_{2}^{\left( 1 \right)}$, $\mathbf{x}_{2}^{\left( 2 \right)}$, $\mathbf{x}_{1}^{\left( 3 \right)}$, $\mathbf{x}_{1}^{\left( 4 \right)}$, $\mathbf{x}_{2}^{\left( 3 \right)}$ and $\mathbf{x}_{2}^{\left( 4 \right)}$ are $L\times$1 vectors of haplotypes; $\boldsymbol{\beta}_{1m}$ and $\boldsymbol{\beta}_{2m}$ are $L\times1$ vectors of marker effects; $L=1951$ represents the length of all markers. When the elements of $\boldsymbol{\beta}_{1m}$ and $\boldsymbol{\beta}_{2m}$​ are 0, it indicates not only that the estimated marker effects for the corresponding population are 0 but also that the markers without polymorphism within the population have an effect of 0.


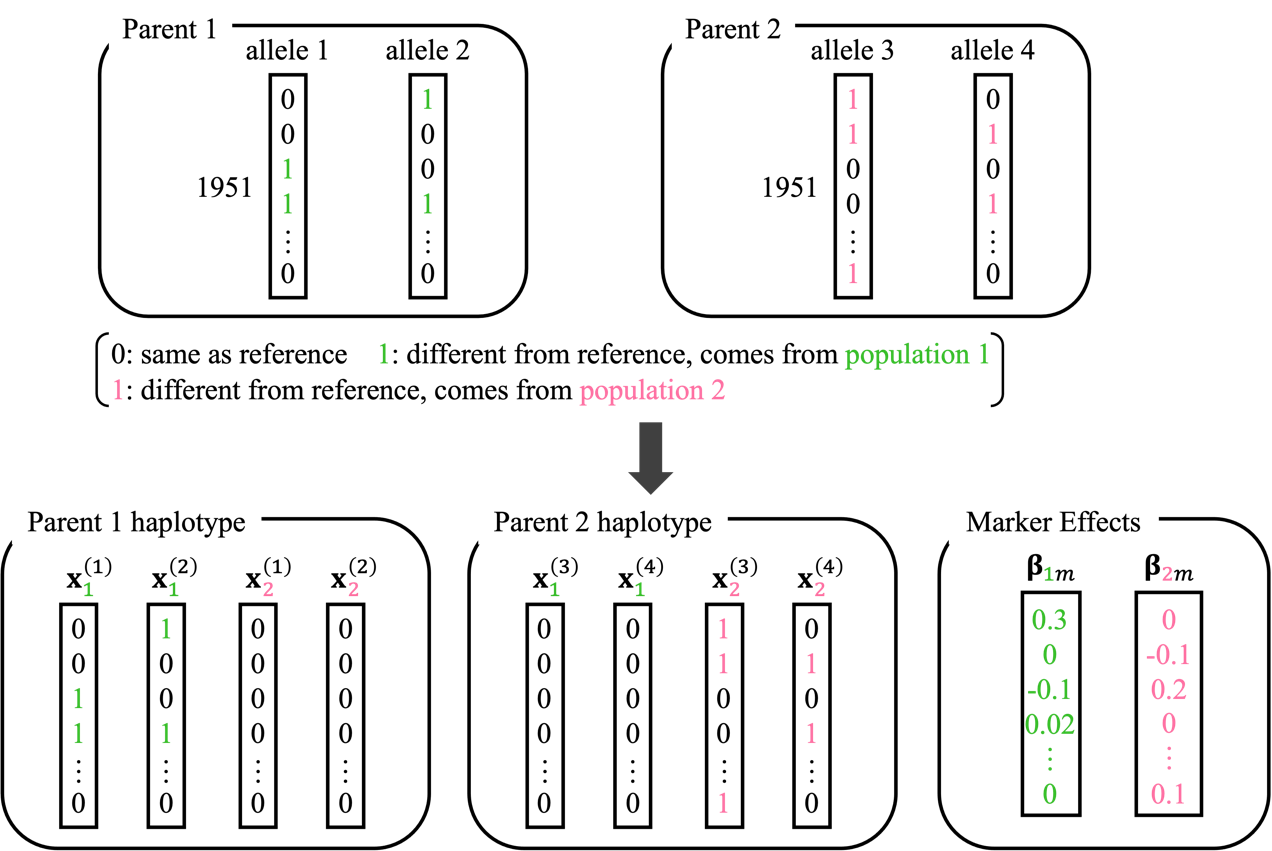
First, we consider the case where the two parents forming the cross pair $k$, Parent 1 and Parent 2, originate from population 1 and population 2, respectively—that is, a case in the first-round cross. The figure below illustrates an example of this case.

Here, the haplotypes of Parent 1 and Parent 2 originate exclusively from their respective populations and are not a mixture of the two populations. Therefore, $\mathbf{x}_{1}^{\left( 1 \right)}$and $\mathbf{x}_{1}^{\left( 2 \right)}$​ correspond to allele 1 and allele 2 of Parent 1, while $\mathbf{x}_{2}^{\left( 3 \right)}$and $\mathbf{x}_{2}^{\left( 4 \right)}$​ correspond to allele 3 and allele 4 of Parent 2. As a result, $\mathbf{x}_{2}^{\left( 1 \right)}$, $\mathbf{x}_{2}^{\left( 2 \right)}$,​ $\mathbf{x}_{1}^{\left( 3 \right)}$ and $\mathbf{x}_{1}^{\left( 4 \right)}$ are all equal to 0.

It is important to note that the value 0 for $\mathbf{x}_{2}^{\left( 1 \right)}$, $\mathbf{x}_{2}^{\left( 2 \right)}$,​ $\mathbf{x}_{1}^{\left( 3 \right)}$ and $\mathbf{x}_{1}^{\left( 4 \right)}$​ does not indicate that these markers match the reference sequence. Rather, it signifies that these markers do not originate from population 1 or population 2.


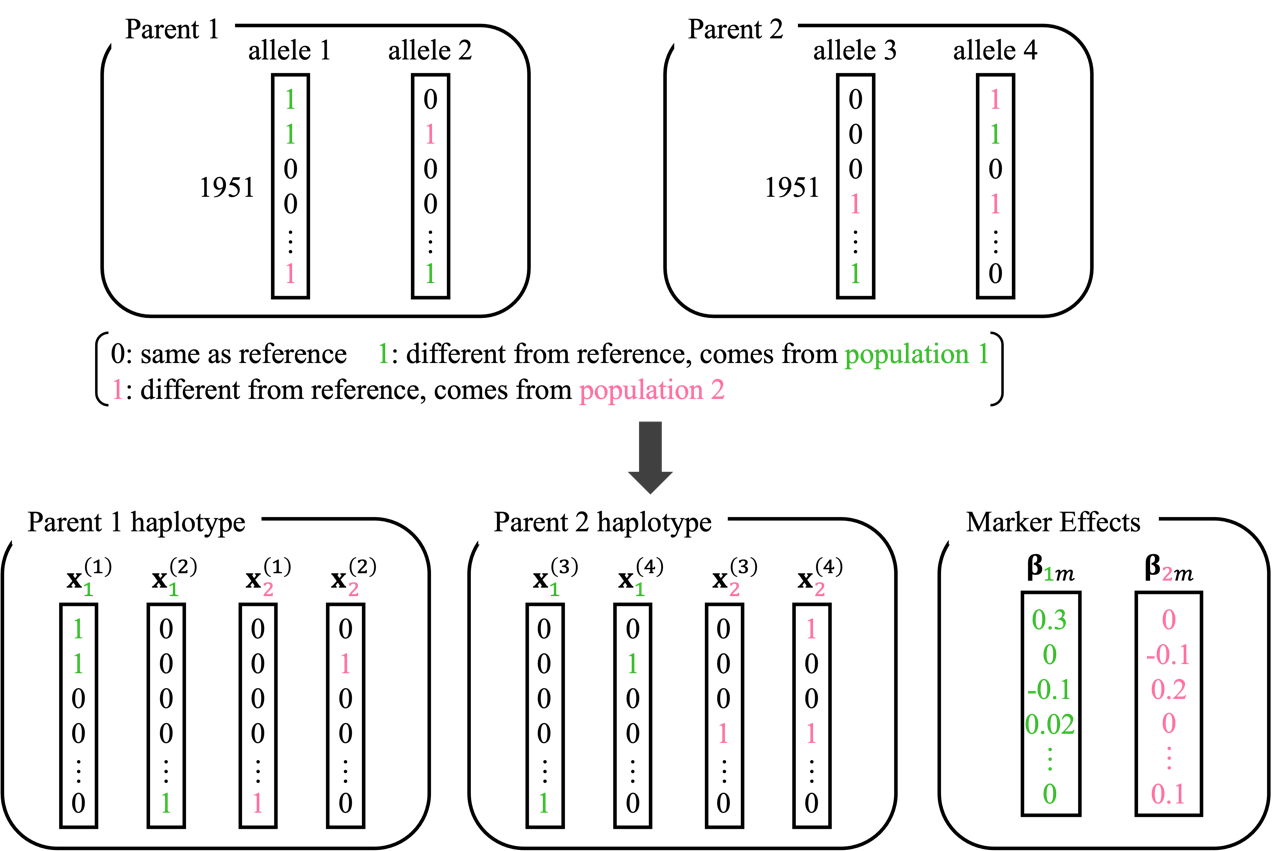
Next, we consider cross pair $k$ in the second-round cross. At this case, the haplotypes of the two individuals, Parent 1 and Parent 2, contain alleles derived from both populations. The figure below illustrates an example of this case.

Here, the sum of each element in $\mathbf{x}_{1}^{\left( 1 \right)}$and $\mathbf{x}_{2}^{\left( 1 \right)}$ corresponds to allele 1 of Parent 1. Since the haplotype of Parent 1 originates from both populations, allele 1 can be divided into markers derived from population 1 and markers derived from population 2, which correspond to $\mathbf{x}_{1}^{\left( 1 \right)}$and $\mathbf{x}_{2}^{\left( 1 \right)}$​, respectively. Similarly, the sum of each element in $\mathbf{x}_{1}^{\left( 2 \right)}$and $\mathbf{x}_{2}^{\left( 2 \right)}$​​ corresponds to allele 2 of Parent 1, the sum of each element in $\mathbf{x}_{1}^{\left( 3 \right)}$and $\mathbf{x}_{2}^{\left( 3 \right)}$​ corresponds to allele 3 of Parent 2, and the sum of each element in $\mathbf{x}_{1}^{\left( 4 \right)}$and $\mathbf{x}_{2}^{\left( 4 \right)}$​ corresponds to allele 4 of Parent 2.
